# Intrinsic Disorder and Phase Separation Coordinate Exocytosis, Motility, and Chromatin Remodeling in the Human Acrosomal Proteome

**DOI:** 10.3390/proteomes13020016

**Published:** 2025-04-28

**Authors:** Shivam Shukla, Sean S. Lastorka, Vladimir N. Uversky

**Affiliations:** 1 Department of Integrative Biology, College of Arts and Sciences, University of South Florida-St. Petersburg, 140 7th Ave. South, St. Petersburg, FL 33701, USA; shuklas1@usf.edu; 2 Department of Molecular Medicine, Morsani College of Medicine, University of South Florida, Tampa, FL 33612, USA; seanlastorka@usf.edu; 3 USF Health Byrd Alzheimer’s Research Institute, Morsani College of Medicine, University of South Florida, Tampa, FL 33612, USA

**Keywords:** proteins, bioinformatics, structure, fertility, reproduction, human sperm, spermatogenesis, acrosome, acrosomal proteins, intrinsic disorder

## Abstract

Intrinsic disorder refers to protein regions that lack a fixed three−dimensional structure under physiological conditions, enabling conformational plasticity. This flexibility allows for diverse functions, including transient interactions, signaling, and phase separation via disorder-to-order transitions upon binding. Our study focused on investigating the role of intrinsic disorder and liquid−liquid phase separation (LLPS) in the human acrosome, a sperm-specific organelle essential for fertilization. Using computational prediction models, network analysis, Structural Classification of Proteins (SCOP) functional assessments, and Gene Ontology, we analyzed 250 proteins within the acrosomal proteome. Our bioinformatic analysis yielded 97 proteins with high levels (>30%) of structural disorder. Further analysis of functional enrichment identified associations between disordered regions overlapping with SCOP domains and critical acrosomal processes, including vesicle trafficking, membrane fusion, and enzymatic activation. Examples of disordered SCOP domains include the PLC-like phosphodiesterase domain, the t-SNARE domain, and the P-domain of calnexin/calreticulin. Protein–protein interaction networks revealed acrosomal proteins as hubs in tightly interconnected systems, emphasizing their functional importance. LLPS propensity modeling determined that over 30% of these proteins are high-probability LLPS drivers (>60%), underscoring their role in dynamic compartmentalization. Proteins such as myristoylated alanine-rich C-kinase substrate and nuclear transition protein 2 exhibited both high LLPS propensities and high levels of structural disorder. A significant relationship (*p* < 0.0001, R² = 0.649) was observed between the level of intrinsic disorder and LLPS propensity, showing the role of disorder in facilitating phase separation. Overall, these findings provide insights into how intrinsic disorder and LLPS contribute to the structural adaptability and functional precision required for fertilization, with implications for understanding disorders associated with the human acrosome reaction.

## 1. Introduction

### 1.1. Spermiogenesis, Structure, Formation, and Function of the Acrosome

Spermatogenesis is the process in which spermatozoa are produced from spermatogonial stem cells. The process takes place in the seminiferous tubules of the testis starting at puberty and continuing for the entirety of an adult male’s life. In the early phases of spermatogenesis, also known as the proliferation phase, spermatogonial stem cells multiply through mitosis and develop into the primary spermatocyte. The primary spermatocytes then undergo meiosis, first dividing into secondary spermatocytes and ultimately resulting in spermatids. The spermatids will then advance to the epididymis where spermiogenesis will take place. During spermiogenesis, the spermatids undergo cytoplasmic rearrangement to form the motile spermatozoa. To form mature spermatozoa, the nucleus elongates, mitochondria condense into the midpiece, the cytoplasm streamlines, the flagellum develops, and the specialized acrosomal organelle forms [1,2].

The acrosome is a sac-like organelle located in the heads of mature spermatozoa and plays a key role in fertilization. It is subdivided into anterior and posterior sections. The membranes of this organelle can be divided into two regions, the outer acrosomal membrane (OAM), which interacts with the plasma membrane, and the inner acrosomal membrane (IAM), which is intricately connected with the nuclear envelope. The lumen of the acrosome, also referred to as the acrosomal matrix, is where its many hydrolytic, proteolytic, receptor, and regulatory proteins are found [1].

The formation of the acrosome starts in the Golgi phase of spermatogenesis as proacrosomal vesicles are released from the Golgi apparatus and fuse together. It then grows and spreads over the anterior half of the nuclear envelope during the cap phase. In the elongation phase, the acrosomal matrix is formed. In the maturation phase, the acrosome takes its characteristic cap-like shape, and the components of the matrix organize themselves into the anterior and posterior sections [1,2].

The main function of the acrosome in fertilization is through the acrosome reaction, binding to and penetrating the zona pellucida (ZP), and fusion of the sperm and egg membrane [1]. The acrosome reaction is calcium ion mediated exocytosis, which releases many hydrolytic and proteolytic enzymes, allowing the sperm to penetrate the oocyte. This reaction is triggered by glycoproteins on the ZP, as well as by the progesterone binding to receptors on the sperm’s plasma membrane, triggering a G protein signal transduction cascade. Once the acrosome reaction has taken place, the protein components are released and begin to interact with the ZP, allowing for the penetration and fusion processes to occur [3].

The decline of male fertility has become a major concern in the 21st century, with sperm dysfunction playing a central role in many infertility cases [4]. Prior studies have demonstrated that IDPs can play essential roles in sperm development, as shown by the IDP SPE− −18 in *Caenorhabditis elegans,* which is required for the assembly of fibrous bodies and proper progression of spermatogenesis [5].

### 1.2. Intrinsic Disorder in Human Proteins

Intrinsically disordered proteins (IDPs) and intrinsically disordered regions (IDRs) represent a unique class of functional proteins that lack a stable three-dimensional structure under physiological conditions [6,7,8,9,10,11,12,13,14]. Unlike globular proteins, which adopt well-defined folds, IDPs/IDRs are characterized by structural plasticity, allowing them to exist as dynamic ensembles of rapidly interconverting conformations (or highly dynamic sets of short-lived structures) [7,9,11]. In fact, this inability to fold is unequally distributed within a protein molecule, with its different parts being under-folded to different degrees. This defines the mosaic structure and astonishing multi-level spatiotemporal heterogeneity of IDP/IDR that represent a complex combination of foldons (independently foldable units), inducible foldons (disordered regions that can (partially) fold at interaction with the binding partners), morphing inducible foldons (disordered regions that can differently fold at interaction with different binding partners), non-foldons (non-foldable protein regions), semi-foldons (regions that are always in a semi-folded form), and unfoldons (ordered regions that have to undergo an order-to-disorder transition to become functional) [15,16]. One should also keep in mind that it is extremely unlikely that different foldons in a given protein would possess identical conformational stability. Furthermore, foldons are known to continually unfold and refold even under native conditions [17,18,19]. As a result, at any given moment, even a well-folded, ordered protein would have a mosaic structure, possessing a set of temporary folded and unfolded foldons [16]. Intrinsic disorder is crucial for structural and functional diversification of proteins contributing to the proteoform concept and the protein structure–function continuum model, where multiple proteoforms (i.e., different molecular forms in which the protein product of a single gene can be found) with different structural features and various functions constitute a highly dynamic conformational ensemble in which a given protein exists [15,20,21].

This inherent flexibility facilitates diverse biological roles and functions, such as in cell signaling, molecular recognition, as well as the regulation of various cellular processes [22,23,24,25,26,27,28,29,30], with the functional repertoire of IDPs/IDRs being typically complementary to functions of ordered proteins and domains [6,10,13,31,32]. The functional versatility of IDPs is linked to their disorder, where differently under-folded pieces of the protein structural mosaic might have well-defined and specific functions [16]. Such structural mosaic defines complex ‘anatomy’ of an IDP that might contain multiple relatively short, differently ordered/disordered functional elements defining its complex molecular ‘physiology’ reflected in its multifunctionality and ability to be involved in interaction with, regulation of, and be controlled by multiple structurally unrelated partners [16,33]. Furthermore, the lack of a fixed structure allows IDPs/IDRs to become engaged in unrestrained interactions with multiple molecules [6,9,10,12,13,14,15,23,34,35,36], often through disorder-to-order transitions upon binding, being even capable of folding differently at interaction with different partners [37,38,39]. This conformational adaptability enhances their binding affinity and specificity, making IDPs important players in cellular processes like protein–protein and protein–nucleic acid interactions [6,40]. Furthermore, IDPs often serve as hubs in interaction networks, and have roles in signaling cascades and regulatory pathways [38,41,42,43,44,45]. Furthermore, structural ‘floppiness’ also defines the ability of IDP/IDR to be controlled and regulated at multiple levels [15,35,40,46], with various post-translational modifications (PTMs) being one of the most important means of such disorder-centered regulation [47,48,49,50,51,52,53,54,55,56,57,58,59,60,61,62,63].

Bioinformatics studies indicated that IDPs and hybrid proteins with ordered domains and IDRs are highly abundant in nature [64,65,66,67,68,69,70,71]. The prevalence of intrinsic disorder in proteomes correlates positively with organismal complexity. Eukaryotic proteomes, for example, exhibit a significantly higher proportion of disorder compared to prokaryotes, with estimates suggesting that more than 60% of eukaryotic proteins contain at least one disordered region. This correlation underscores the evolutionary advantage and function of disorder in facilitating complex regulatory mechanisms and molecular interactions essential for multicellular life [72,73,74,75,76,77,78,79,80,81].

Despite their functional benefits, the unstructured nature of IDPs renders them susceptible to misfolding and aggregation under dysregulated conditions. This susceptibility is shown in numerous human diseases, including neurodegenerative disorders, cancer, and cardiovascular diseases [82,83,84,85,86,87,88,89,90,91,92,93,94,95,96,97,98,99,100,101,102,103]. For example, amyloidogenic IDPs such as α-synuclein and tau are central to the pathology of Parkinson’s disease and Alzheimer’s disease, respectively [104,105]. The duality of IDPs, as both functional and potentially pathological entities, highlights the importance of understanding the mechanisms underlying their behavior.

Moreover, the interplay between order and disorder in proteins extends their functional use. As was already emphasized, many IDPs exhibit hybrid structural features, comprising ordered domains alongside disordered regions. This arrangement allows for spatial and temporal modulation of protein activity, as seen in proteins involved in transcriptional regulation and signal transduction [106]. The coupling of intrinsic disorder with specific post-translational modifications further increases their functional diversity and adaptability, showing their significance in cellular homeostasis and evolution.

The study of IDPs is important, as it provides insights into the principles of protein structure and function, challenging the traditional structure–function paradigm. Their intrinsic disorder, while structurally unconventional, is an essential part of their biological utility, from mediating dynamic interactions to organizing cellular architecture through phase separation. As the roles of IDPs in health and disease continue to be studied, more work remains to understand their structural and functional biology and biophysical properties.

### 1.3. Spontaneous Liquid–Liquid Phase Separation

Intrinsically disordered proteins (IDPs) play a central role in liquid–liquid phase separation (LLPS) [107,108,109,110,111,112,113,114,115,116], a process where biomolecules condense into dense, protein- and nucleic acid-rich droplets that are distinct from the surrounding dilute phase. These phase-separated assemblies, or biomolecular condensates, contribute to cellular organization by forming membraneless organelles, such as nucleoli, stress granules, and Cajal bodies [117,118]. The ability of IDPs to drive LLPS is dictated by their unique sequence characteristics, including charge distribution, hydropathy, and aromatic content, as well as their enrichment in low-complexity domains and intrinsically disordered regions. These features cause multivalent interactions through weak, transient associations that promote the assembly of dense biomolecular condensates.

The sequence composition of IDPs influences LLPS. For instance, the patterning of charged residues within an IDP, such as the distribution of positively and negatively charged amino acids, can modulate phase behavior. Sequences with block-like charge arrangements exhibit larger phase separation windows than those with randomly distributed charges. This relationship shows the importance of electrostatic interactions in IDP-driven coacervation [119,120]. Similarly, aromatic and hydrophobic residues in IDPs can contribute to phase separation by mediating π-π stacking or hydrophobic interactions within condensates [118].

IDPs associated with LLPS serve diverse biological functions. For instance, phase-separated droplets act as reservoirs for biomolecules, regulate biochemical reactions by concentrating reactants, and serve as structural scaffolds for assembling macromolecular complexes [121,122]. IDP-mediated phase separation also plays a critical role in pathological contexts. Aberrant LLPS has been implicated in the formation of amyloid-like aggregates in diseases such as amyotrophic lateral sclerosis (ALS) and frontotemporal dementia (FTD), where phase-separated intermediates composed of IDPs, such as FUS and TDP-43, transition to pathological solid states [123]. Although these examples stem from neurodegenerative diseases, similar dysregulation of phase separation and intrinsic disorder in the acrosome could potentially contribute to sperm dysfunction and male infertility.

From a biophysical perspective, the ability of IDPs to undergo LLPS is regulated by environmental conditions, including pH, salt concentration, and temperature. These factors change the physicochemical properties of IDPs, such as their net charge and hydrophobicity, thereby influencing the phase diagram of their condensates [120]. Advanced computational and experimental approaches, including molecular dynamics simulations and fluorescence microscopy, have been employed to understand the molecular principles regulating IDP-mediated LLPS. Field-theoretic simulation (FTS) approaches, for example, have provided insights into how charge patterning and sequence variation dictate phase behavior in polyampholyte IDPs [119,120].

Overall, the relationship between IDPs and LLPS highlights a mechanism of cellular organization that operates beyond the classical paradigms of compartmentalization. The ability of IDPs to phase-separate, forming dynamic and tunable assemblies, highlights their biological importance and function, as well as their potential as research targets in therapeutic interventions for modulating biomolecular condensates.

## 2. Materials and Methods

### 2.1. Compilation of Human Acrosomal Proteins and Visualization

On 19 September 2024, proteins related to the human acrosome were compiled from UniProt (https://www.uniprot.org (accessed 19 September 2024)) using the search term ‘acrosome OR acrosomal’ and filtering the results to only include SwissProt reviewed, human entries. UniProt IDs, protein names, gene names, sequences in FASTA format and functional information were extracted for each protein and used to build the dataset.

If available, experimentally derived protein structures were obtained from the Protein Data Bank (https://www.rcsb.org (accessed 19 September 2024)). If they were not available, predicted protein structures were obtained from the AlphaFold Protein Structure Database (https://alphafold.ebi.ac.uk (accessed 19 September 2024)).

**Figure 1:**
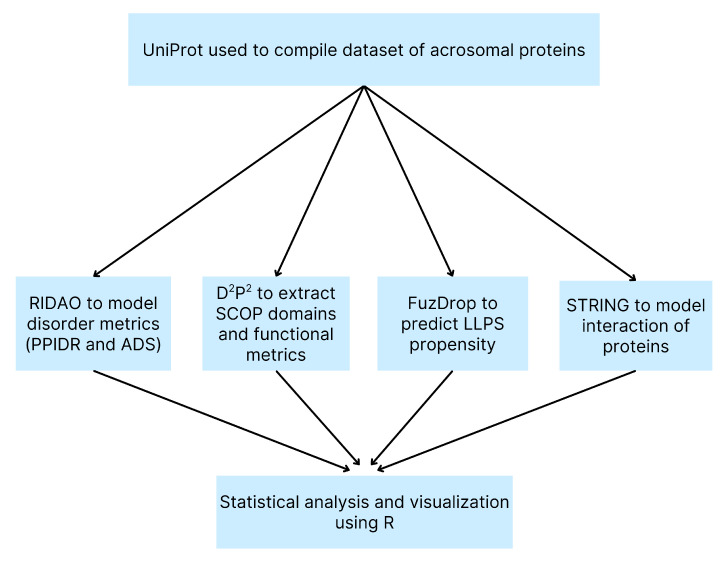
Flowchart of Computational Methods. Overview of the bioinformatics pipeline used to analyze the human acrosomal proteome. A dataset of acrosomal proteins was compiled from UniProt and analyzed using RIDAO to predict intrinsic disorder (PPIDR and ADS), D²P² to identify SCOP domains and functional features, FuzDrop to evaluate LLPS propensity, and STRING to model protein-protein interactions. Outputs from all tools were integrated and statistically analyzed using R for visualization and interpretation.

An overview of our computational methods is shown in a flowchart (Figure 1) for clarity.

### 2.2. Computational Prediction of Intrinsic Disorder in Human Acrosomal Proteins

The prediction of intrinsic disorder in the dataset of proteins was conducted using six widely recognized computational models, accessed via the Rapid Intrinsic Disorder Analysis Online RIDAO platform (https://ridao.app (accessed 24 September 2024)) [124]. These models include PONDR^®^ VLXT, which integrates three neural networks designed to predict disorder across the full length of a protein sequence, combining results from terminal and internal region predictors [125]. PONDR^®^ VL3, which focuses on long disordered regions and uses multiple neural networks to generate predictions [126]. PONDR^®^ VSL2B which uses support vector machines (SVMs) and is optimized for both short and long disordered regions [127]. The IUPred model, which is different from the previous machine learning models, estimates disorder by evaluating inter-residue interaction energies, relying on energy-based potentials to predict regions unlikely to form stable structures, and which is run in two modes, to predict short and long IDRs [128,129]. Finally, PONDR-FIT is an artificial neural network (ANN) meta-predictor that combines outputs from multiple disorder prediction models [130]. These models were aggregated by RIDAO [124] to provide a comprehensive disorder prediction profile for each acrosomal protein.

Protein disorder was evaluated using two metrics obtained from each of the six models: the average disorder score (ADS) and the percentage of predicted intrinsically disordered residues (PPIDR). For each protein, the ADS was calculated by taking the arithmetic mean of all residue-specific disorder scores. The PPIDR was determined by counting the number of residues with a disorder score greater than 0.5, dividing this by the total number of residues, and expressing the result as a percentage. Using these metrics, we classified proteins into distinct categories. Based on ADS, proteins were considered highly ordered if their score was below 0.25, moderately disordered if between 0.25 and 0.5, and highly disordered if 0.5 or higher. Similarly, using PPIDR, proteins were classified as highly ordered (PPIDR < 10%), moderately disordered (10% ≤ PPIDR < 30%), or highly disordered (PPIDR ≥ 30%). These categories were based on established baselines of disorder found in the literature.

Then, RIDAO was further used to obtain the outputs from two binary predictors, the charge-hydropathy (CH) plot [11] and the cumulative distribution function (CDF) plot [131], which were combined to generate a CH-CDF phase diagram [132,133,134]. This allowed for the classification of proteins based on their location within the CH-CDF phase space. Proteins in Quadrant I can be categorized as ordered (predicted to be ordered by both models), Quadrant III can be categorized as native coils or pre-molten globules (disordered by both methods), Quadrant II can be categorized as hybrid proteins or putative native molten globules (ordered by CH but disordered by CDF), and Quadrant IV can be categorized as proteins that are disordered according to the CH plot but ordered by the CDF plot [132].

### 2.3. Impact of Intrinsic Disorder on Function in Human Acrosomal Proteins

Disorder predictions and related functional annotations for the dataset of proteins were retrieved from the D^2^P^2^ database, which provides predictions for certain indexed proteins from fully sequenced genomes [135]. D^2^P^2^ aggregates results from several well-established computational per-residue disorder prediction models, including PONDR^®^ VLXT [125], PONDR^®^ VSL2B [127], IUPred (both short and long forms) [128,129], PrDOS [136], PV2, and three variants of ESpritz (NMR, DisProt, and X-ray) [137]. The predicted locations of disordered regions are displayed as nine colored bars in the D^2^P^2^ visual interface, each representing a different model. In addition to individual predictions, D^2^P^2^ also presents a consensus view of disorder across all predictors using a blue-green-white bar, where blue indicates regions where the disorder predictions intersect the SCOP domain prediction and green indicates regions that represent disorder that is not found within a predicted SCOP domain [135]. Above this consensus bar, two lines with numbered, colored bars show the predicted locations of SCOP (Structural Classifications of Proteins) domains [138,139], identified by the SUPERFAMILY predictor [140]. Empirically confirmed post-translational modifications (PTMs), identified by the PhosphoSitePlus platform [141], are shown as differently colored circles at the bottom of the plot. Functional annotations of disordered regions are also provided, such as the predicted disorder-based binding sites (MoRF regions) identified by the ANCHOR algorithm [142,143], which are displayed as yellow patterned bars.

Acrosomal proteins in the dataset were classified based on the presence of at least one residue with 75% consensus for predicted disorder and at least one strong SCOP domain hit via D^2^P^2^. Proteins meeting these criteria were further analyzed to identify instances of overlap between consensus disordered regions and SCOP domains, which may suggest potential functional implications of the intrinsic disorder. The superfamilies of the SCOP domains affected by disordered regions were further analyzed based on the number of residues impacted and to determine whether certain SCOP superfamilies were more frequently affected by disorder within proteins associated with the acrosome.

### 2.4. Gene Ontology Enrichment Analysis of Acrosomal Proteins

Gene Ontology (GO) enrichment analysis was conducted using the g:GOSt tool, part of the g:Profiler (https://biit.cs.ut.ee/gprofiler/gost (accessed 26 September 2024)), to understand the biological processes, molecular functions, and cellular components associated with proteins that exhibited overlap between SCOP domains and regions of predicted intrinsic disorder. The analysis was performed specifically on proteins with at least one SCOP functional domain and 75% consensus for disordered residues, as identified through the D^2^P^2^ platform.

The tool g:GOSt looks at functional enrichment analysis by mapping input proteins to known GO terms and testing for overrepresentation of specific GO categories in the proteins of interest. The tool uses a hypergeometric test to assess enrichment, and statistical significance is controlled using the Benjamini–Hochberg false discovery rate (FDR) correction to minimize type I errors. For the purposes of our study, g:GOSt was used to categorize proteins into three main GO functional domains: Biological Process (BP) (e.g., cellular activities, reproductive functions), Molecular Function (MF) (e.g., binding activity, enzymatic activity), and Cellular Component (CC) (e.g., membrane regions, subcellular structures). Only significantly enriched terms with an adjusted FDR < 0.05 were considered in the final analysis. In addition to GO terms, g:GOSt also yielded information on KEGG (Kyoto Encyclopedia of Genes and Genomes) pathways, Reactome pathways, and other functional categories, including protein complexes and human phenotype ontology. This approach identified important biological roles and functional characteristics associated with disorder in SCOP domains. This will allow for insight into potential functional implications of disordered regions in acrosomal proteins.

### 2.5. Intractability Analysis of Human Acrosomal Proteins

The interactions of the 250 acrosomal proteins were retrieved and visualized using Search Tool for the Retrieval of Interacting Genes (STRING) (https://string-db.org (accessed 6 October 2025)). STRING utilizes experimentally validated information and predictions to generate models of protein–protein interactions (PPI) [144]. Proteins in the network maps are represented as nodes and the interactions are represented as edges. The model’s interactions are broken up into seven categories, each represented by different color edges. Known interactions are represented by light blue for evidence from a database and purple represents experimentally determined interactions. For predicted interactions, green represents proteins in the same gene neighborhood, red represents gene fusion, blue represents gene co-occurrence, black represents co-expression, light purple represents protein homology, and green represents text mining evidence [144].

String was utilized to generate three types of models: a PPI network between the 250 human acrosomal proteins, a PPI network centered on the 250 human acrosomal proteins, and individual networks centered on individual human acrosomal proteins. Models were generated at 4 different confidence intervals 0.15 (low confidence), 0.4 (medium confidence), 0.7 (high confidence), and 0.9 (highest confidence) and were run with a maximum of 500 interactions in the first shell for the global and the individual PPI networks [106,145]. For the PPI networks centered on individual human acrosomal proteins, a confidence threshold of 0.4 was used to maintain consistency across all networks and to align with thresholds commonly used in previous studies [145]. With each model, STRING embedded processes generate network statistics, such as the number of nodes, the average node degree which represents the average number of interactions per protein, the average clustering coefficient which is a number between 0 and 1 (0 represents each neighboring protein which are only connected to one protein, while 1 represents each neighboring protein which are interacting with each other), and a PPI enrichment *p*-value, which is the probability of having more interactions than predicted for a random set of proteins [106,144,145,146].

### 2.6. Liquid–Liquid Phase Separation Propensity in Human Acrosomal Proteins

To evaluate the potential of the acrosomal proteins for liquid–liquid phase separation (LLPS), we employed FuzDrop, a computational tool that predicts LLPS propensity based on protein sequence features [147,148]. FuzDrop uses sequence complexity and specific interaction motifs, such as cation-π or hydrophobic contacts, to determine the likelihood of droplet state formation in proteins. The LLPS analysis provided insights into sequence regions that support non-specific interactions crucial for droplet formation.

For each protein, we calculated the probability of forming a droplet state (probability of liquid–liquid phase separation; p_LLPS_) through FuzDrop [147,148]. Proteins with a p_LLPS_ of ≥0.60 were classified as droplet-drivers, meaning they can independently undergo LLPS. On the other hand, proteins with p_LLPS_ < 0.60 were classified as droplet-clients, which need additional interactions to partition into condensates. These droplet-clients may still participate in LLPS through specific droplet-promoting regions, defined by consecutive residues with high probability values (p_DP_ ≥ 0.60). This analysis allowed us to distinguish between proteins that can spontaneously phase-separate and those dependent on cellular or environmental contexts.

Next, droplet-promoting regions were identified based on residue-level droplet-promoting probabilities (p_DP_), with regions classified by at least five consecutive residues scoring pDP ≥ 0.60. These regions are considered main contributors to phase separation propensity and were used to assess LLPS potential, including within proteins classified as droplet-clients. We then assessed the presence of aggregation hot-spots, which are residues with high droplet-promoting probabilities (p_DP_ ≥ 0.60) and high interaction mode divergence (S_BIND_ ≥ 2.2). These hot-spots indicate a predisposition to aggregation within condensates, especially under conditions that promote a shift from liquid-like to solid-like states. The analysis also included cellular context-dependence, with S_BIND_ values calculated to show the potential for residues to switch between binding modes based on cellular environment. Residues with S_BIND_ ≥ 2.25 were classified as context-dependent interaction zones, which means that the binding mode diversity might influence LLPS behavior and potential of aggregation.

To analyze the relationship between the probability of liquid–liquid phase separation (p_LLPS_) and the percentage of predicted intrinsically disordered residues (PPIDR) from three models (PONDR^®^ VXLT, PONDR^®^ VL3, and PONDR^®^ VSL2B), we employed a regression model. Due to the presence of boundary values in p_LLPS_ (0 and 1), we adjusted these values to avoid computational issues by applying small corrections (0 replaced with 10^−6^, and 1 replaced with 1 10^−6^). Then, a Box–Cox transformation was used on the adjusted p_LLPS_ values to normalize the distribution, with an optimal lambda of 0.0606 determined empirically. This transformation resulted in the creation of a new variable, transformed p_LLPS_, which we used as the response variable in the model.

Then, we fitted a second-degree polynomial regression model to assess the relationship between transformed p_LLPS_ and PPIDR, capturing any potential non-linear patterns in the data. We then conducted diagnostic analyses to evaluate the assumptions underlying our regression model. A Residuals vs. Fitted plot was generated to assess linearity, allowing us to verify that residuals displayed no systematic pattern across fitted values. To evaluate the normality of residuals, we used a Q–Q plot, comparing the distribution of residuals against a theoretical normal distribution. Then, we used a Scale-Location plot to assess homoscedasticity, to ensure that the residuals maintained a constant level of variance across the fitted values. Lastly, we assessed the Residuals vs. Leverage plot to identify any potential influential data points with high leverage or large residuals that might disproportionally affect the model.

### 2.7. Data Analysis via R

All data analysis was conducted in R (version 4.4.2) with RStudio (version 2024.04.2+764) by employing a wide range of packages to process, visualize, and analyze the acrosomal protein dataset. Data manipulation was performed using the packages dplyr and tidyr, which allowed for the merging of datasets, reshaping data from wide to long formats, and calculating summary statistics. Importation and management of CSV and Excel data files was completed using the packages readr and readxl, respectively. For correlation analysis and model fitting, R’s built-in stats package functions were used. The ggplot2 package was used to create various visualizations, including scatter plots, boxplots, histograms, and pie charts. The package gridExtra was applied for arranging multiple plots into a single figure. Additional enhancements to these plots, such as label placement in scatter plots, were achieved using the package ggrepel. To create formatted tables of results, the kableExtra package was utilized.

## 3. Results

### 3.1. Compilation of Human Acrosomal Proteins and Visualization

**Figure 2:**
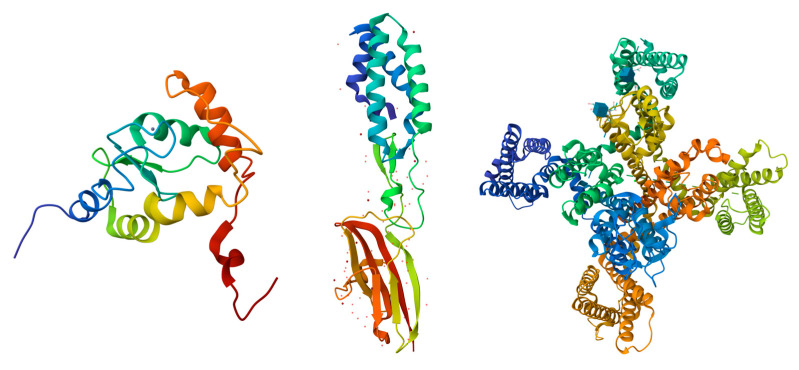
Structural Representations of Selected Proteins. From left to right, the structures shown are: Arf-GAP domain and FG repeat-containing protein 1 (obtained via Solution NMR; PDB ID: 2D9L; [149,150], Sperm acrosome membrane-associated protein 6 (obtained via X-ray diffraction; PDB ID: 7TA2; [151]), and Voltage-dependent T-type calcium channel subunit alpha-1H (obtained via Cryo-EM; PDB ID: 7WLJ; [152]). Each structure is colored in a rainbow gradient from the N-terminus (blue) to the C-terminus (red), highlighting the secondary structural features, including α-helices, β-strands, and loops.

**Figure 3:**
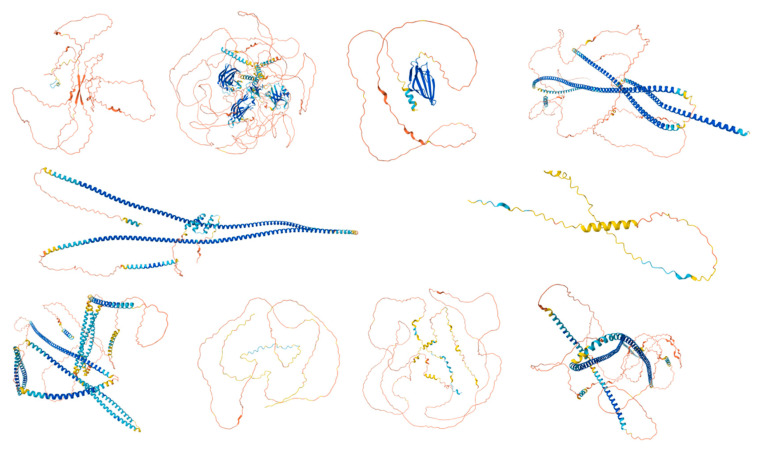
Predicted Structures of the Top 10 Most Disordered Proteins. AlphaFold was used to model the predicted structures of the 10 most disordered proteins in our dataset, creating a structural portrait gallery. Proteins are displayed by UniProt ID from left to right, starting with the top row: Calcium-binding and spermatid-specific protein 1 (UniProt ID: Q96KC9), regulating synaptic membrane exocytosis protein 1 (UniProt ID: Q86UR5), acrosomal protein SP-10 (UniProt ID: P26436), TATA element modulatory factor (UniProt ID: P82094), Golgin subfamily A member 1 (UniProt ID: Q92805), nuclear transition protein 2 (UniProt ID: Q05952), coiled-coil domain-containing protein 136 (UniProt ID: Q96JN2), myristoylated alanine-rich C-kinase substrate (UniProt ID: P29966), Cylicin-1 (UniProt ID: P35663), and centrosomal protein of 131 kDa (UniProt ID: Q9UPN4). Colors represent the predicted local distance difference test (p_LDDT_) scores: very high confidence (p_LDDT_ > 90, dark blue), high confidence (90 > p_LDDT_ > 70, light blue), low confidence (70 > p_LDDT_ > 50, yellow), and very low confidence (p_LDDT_ < 50, orange).

The UniProt database search yielded a total of 250 proteins associated with the human acrosome, which were used for the present study. Representative examples of these 250 proteins were selected and are shown in Figure 2, which shows illustrative examples of experimentally derived protein structures obtained from the Protein Data Bank, and Figure 3, which displays predicted structures of the 10 most disordered proteins obtained from the AlphaFold Protein Structure Database.

### 3.2. Computational Prediction of Intrinsic Disorder in Human Acrosomal Proteins

**Figure 4:**
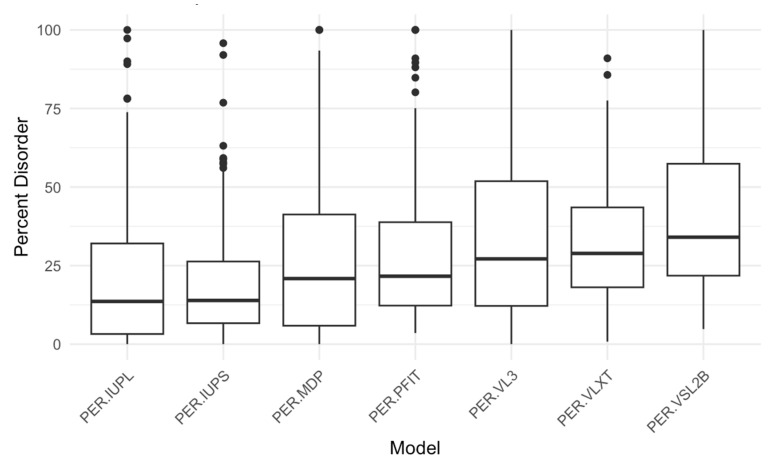
Percent Disorder Across Prediction Models. This boxplot compares the distribution of percent disorder (PER) predicted by seven different models: IUPred long, IUPred short, mean disorder propensity (MDP), PONDR-FIT, PONDR^®^ VL3, PONDR^®^ VLXT, and PONDR^®^ VSL2B (PER-IUPL, PER-IUPS, PER-MDP, PER-PFIT, PER-VL3, PER-VLXT, and PER-VSL2B, respectively). Each box represents the interquartile range (IQR), with the median marked by a horizontal line and outliers shown as individual points. The PER-VSL2B model predicts the highest percent disorder overall, while models such as PER-IUPL and PER-IUPS show lower median values, indicating variability in disorder prediction across different tools.

Using RIDAO, we generated disorder predictions for our 250 acrosomal proteins, analyzing each protein across the models. Key descriptive statistics for 3 of the models (PONDR^®^ VXLT, PONDR^®^ VSL2B, and PONDR^®^ VL3) disorder scores (minimum, mean, median, and maximum) are summarized in Table 1. For example, as per PONDR^®^ VL3, the mean PPIDR score across all proteins was 33.37, with values ranging from 0 to 100, indicating a wide variation in intrinsic disorder across our dataset. Similarly, based on corresponding ADS values, the PONDR^®^ VXLT model exhibited the lowest minimum score (0.0602), while PONDR^®^ VSL2B had the highest maximum score (0.9907). A series of boxplots to show percent disorder values across our dataset between different models was also created (Figure 4).

These findings indicate that the degree of intrinsic disorder in acrosomal proteins spans a broad spectrum, potentially reflecting diversity of their disorder-based functionalities. Proteins with low disorder scores are likely to be involved in roles requiring structural stability, such as enzymatic catalysis, transport, scaffolding, or maintaining the architecture of the acrosome, whereas those with high disorder scores may contribute to dynamic processes, such as vesicle trafficking or molecular recognition. The observed range suggests a selective advantage for both ordered and disordered structures within the acrosomal proteome, enabling the acrosome to fulfill its complex roles in fertilization.

**Figure 5:**
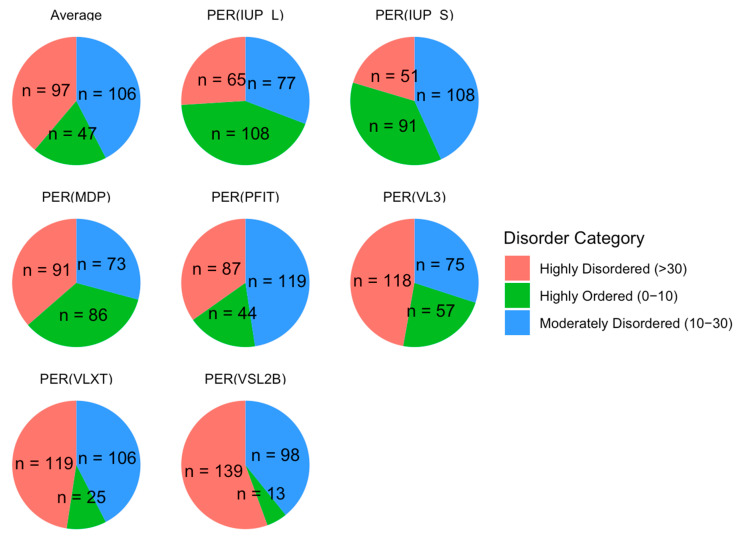
Protein Disorder Categories by Model. This figure shows the distribution of proteins categorized into three disorder classes across multiple prediction models. Proteins are classified as highly disordered (>30% disordered residues, red), moderately disordered (10–30% disordered residues, blue), and highly ordered (0−10% disordered residues, green). Each pie chart corresponds to a specific prediction model. The numbers (n) represent the count of proteins within each disorder category for the respective model.

We classified proteins by their PPIDR values into three categories: highly ordered (0−10%), moderately disordered (10−30%), and highly disordered (>30%). As illustrated in Figure 5, this classification showed that 97 proteins were highly disordered, 106 were moderately disordered, and 47 were highly ordered on average across all models. These proportions emphasize a skew toward moderate and high levels of disorder in the acrosomal protein dataset. This distribution aligns with previous findings in other specialized cellular structures, such as the nucleolus and synaptic vesicles [153,154], where intrinsic disorder is prevalent and facilitates rapid assembly and disassembly of macromolecular complexes. The high proportion of moderately and highly disordered proteins in the acrosomal dataset may similarly reflect an adaptation for flexible interactions required during the fertilization process.

**Figure 6:**
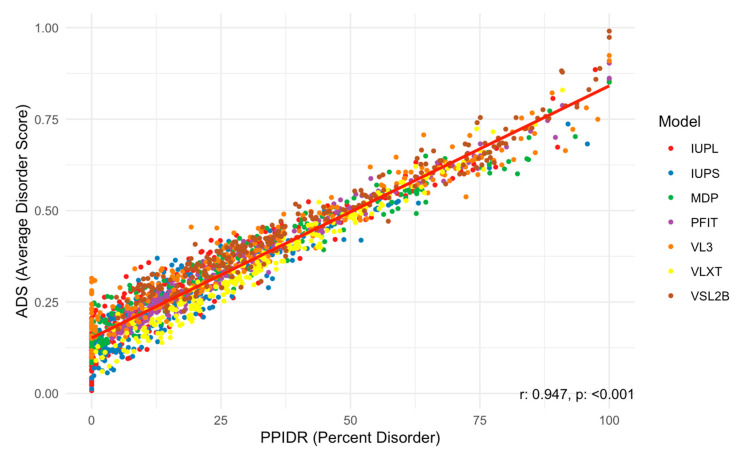
Scatter Plot of PPIDR vs. ADS Across All Models Analyzed. This scatter plot shows the relationship between PPIDR (percent disorder) and ADS (average disorder score) across multiple prediction models. Each dot represents a protein, colored by the respective disorder prediction model IUPred long, IUPred short, MDP, PONDR-FIT, PONDR^®^ VL3, PONDR^®^ VLXT, and PONDR^®^ VSL2B (IUPL, IUPS, MDP, PFIT, VL3, VLXT, and VSL2B). A strong positive correlation is observed, with a correlation coefficient of r = 0.947 and a statistically significant *p*-value (*p* < 0.001), indicating a strong linear relationship between percent disorder and average disorder score.

Next, we examined the relationship between the PPIDR and ADS scores to better understand correlations between these two disorder metrics. As shown in Figure 6, the scatter plot of PPIDR vs. ADS reveals a consistent trend (*p* < 0.001) with higher ADS values generally associated with increased PPIDR, suggesting a relationship between these metrics across the majority of proteins. This trend further supports the idea that disorder−related properties, such as extended conformations or binding promiscuity, are integral to the function of many acrosomal proteins. Notably, proteins with high ADS values may exhibit increased potential for transient interactions, such as binding to sperm or oocyte receptors, or participating in acrosomal vesicle fusion.

**Figure 7:**
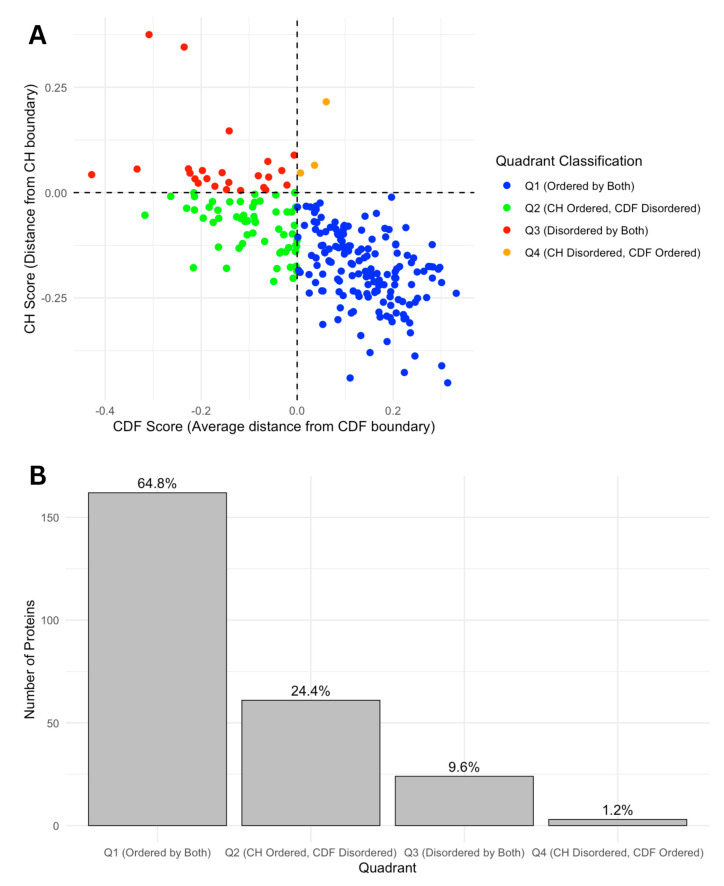
(**A**) CH-CDF plot of intrinsic disorder status. This scatter plot displays the relationship between CH scores (distance from CH boundary) and CDF scores (average distance from CDF boundary), classifying proteins into four quadrants based on their intrinsic disorder status. Quadrants are defined as Q1 (Ordered by both CH and CDF boundaries, blue), Q2 (CH Ordered, CDF Disordered, green), Q3 (Disordered by both CH and CDF boundaries, red), and Q4 (CH Disordered, CDF Ordered, orange). This classification highlights the agreement or disagreement between CH and CDF disorder predictions. (**B**). Number and ratio of proteins in each quadrant. The bar chart shows the distribution of proteins across four quadrants based on CH-CDF disorder classification. The percentages on the top of each bar represent the percent of total proteins in that category.

We further categorized our proteins based on Charge-Hydropathy (CH) and Cumulative Distribution Function (CDF) scores. Figure 7A shows a scatter CH vs. CDF graph, whereas Figure 7B presents a bar chart showing quadrant distributions, where each quadrant represents a different prediction alignment between CH and CDF: Q1 (lower-right quadrant) includes proteins predicted as ordered by both CH and CDF; Q2 (lower-left quadrant) includes those ordered by CH but disordered by CDF; Q3 (upper-left quadrant) includes proteins disordered by both predictors; and Q4 (upper-right quadrant) represents those disordered by CH but ordered by CDF. This quadrant analysis provides a structured view of how each protein’s disorder may vary based on predictor alignment.

The quadrant distribution offers additional insight into the complexity of disorder prediction and its functional implications. Q3 proteins, predicted as disordered by both CH and CDF, align well with the high levels of intrinsic disorder observed in the acrosomal proteome and likely represent proteins with a high degree of flexibility essential for dynamic processes like vesicle fusion or enzymatic activation. In contrast, Q1 proteins, which are ordered by both predictors, may play more static structural roles, supporting the acrosomal matrix or organizing its architecture. Q2 and Q4 proteins, where predictor alignments diverge, present a middle ground. Proteins in Q2 (ordered by CH but disordered by CDF) may exhibit conditional disorder, becoming functional only under specific physiological conditions such as changes in pH, ion concentration, or binding partner availability. Similarly, Q4 proteins (disordered by CH but ordered by CDF) may represent cases where localized order is embedded within a largely disordered protein, enabling dual functionalities. These nuances highlight how prediction alignments offer additional perspectives on the roles of intrinsic disorder in the acrosomal proteome.

These findings suggest the critical role of intrinsic disorder in acrosomal function. The acrosome, as a specialized organelle, must balance structural stability with dynamic adaptability during sperm capacitation, the acrosome reaction, and fertilization. Our analysis highlights how intrinsic disorder in acrosomal proteins contributes to this balance, enabling complex processes such as enzymatic activation, membrane remodeling, and molecular recognition. These results provide a foundation for future studies investigating how intrinsic disorder in acrosomal proteins contributes to reproductive biology and potential implications for fertility disorders.

### 3.3. Impact of Disorder on Function in Human Acrosomal Proteins

**Figure 8:**
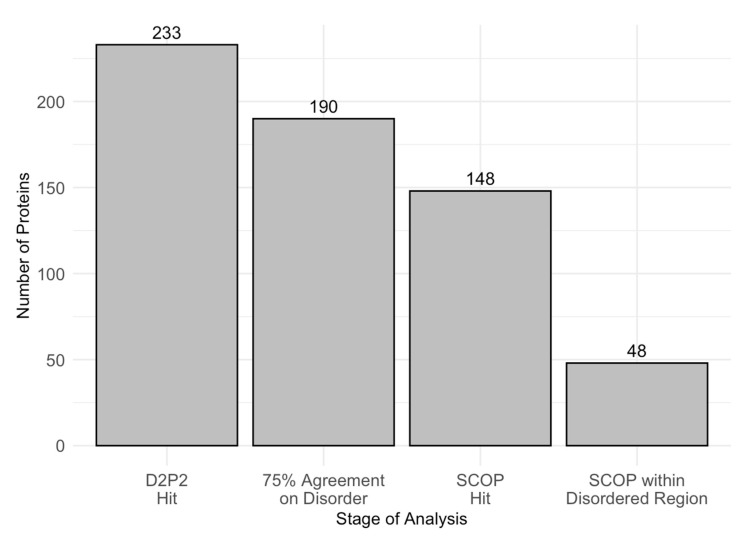
Number of Proteins at Each Analysis Stage. This bar chart displays the number of proteins retained through successive stages of analysis. The stages include D2P2 hits (233 proteins), proteins with 75% agreement on disorder prediction (190 proteins), SCOP hits (148 proteins), and SCOP domains located within disordered regions (48 proteins). This stepwise filtering highlights the progressive narrowing of the dataset to identify proteins with structural disorder within SCOP domains overlap.

To further examine intrinsic disorder in acrosomal proteins, we utilized D^2^P^2^ to identify proteins with disordered residues in SCOP functional domains, using a 75% agreement threshold. Out of 233 proteins initially analyzed, 190 met the high−confidence disorder threshold, and 148 were found to contain identifiable SCOP domains. Among these, 48 proteins showed overlap between disordered regions and SCOP domains. Figure 8 displays the number of proteins progressing through each analysis stage, ultimately identifying the 48 proteins with SCOP-associated disordered regions. Furthermore, Table 2 provides a comprehensive list of SCOP superfamilies affected by disorder, highlighting those that are commonly disordered and frequently observed across different proteins. The identification of these SCOP-associated disordered regions underscores the selective pressure for disorder in specific functional domains, which may play a crucial role in the specialized functions of acrosomal proteins.

The most common SCOP superfamilies affected by disorder included domains associated with structural flexibility and protein–protein interactions. This prevalence supports the hypothesis that intrinsic disorder is a key driver of functional adaptability, particularly in dynamic environments like the acrosome. Several superfamilies appeared multiple times across different proteins, indicating a widespread occurrence of intrinsic disorder within certain functional domains. Notably, the PLC-like phosphodiesterase superfamily showed the highest incidence of disorder, with 111 disordered residues across multiple proteins (1-phosphatidylinositol 4,5-bisphosphate phosphodiesterase delta-4 (UniProt ID: Q9BRC7) and 1-phosphatidylinositol 4,5-bisphosphate phosphodiesterase beta-1 (UniProt ID: Q9NQ66)). This observation suggests that intrinsic disorder within the PLC-like domains may facilitate enzymatic flexibility and regulation during membrane-related signaling processes, which are vital in the context of sperm-egg recognition and fertilization.

The t-SNARE proteins superfamily also exhibited significant disorder, with 73 residues disordered across proteins such as Syntaxin-2 (UniProt ID: P32856), Syntaxin-1B (UniProt ID: P61266), and Syntaxin-1A (UniProt ID: Q16623). The disordered regions in these proteins are likely critical for vesicle docking and membrane fusion, processes essential for acrosomal exocytosis. Additionally, the Concanavalin A-like lectins/glucanases superfamily exhibited 40 disordered residues in both Calreticulin (UniProt ID: P27797) and E3 ubiquitin-protein ligase TRIM36 (UniProt ID: Q9NQ86), suggesting a role in mediating dynamic interactions during immune-related functions or protein ubiquitination pathways in acrosomal environments.

In our dataset, several acrosomal proteins exhibited multiple SCOP domains within disordered regions, indicating complex structural architectures. For example, Calreticulin (UniProt ID: P27797) contained both a C-type lectin domain and a collagen-like domain within disordered regions, facilitating its role in pathogen recognition and immune response. This structural configuration likely enhances the ability of Calreticulin to engage in multiple interactions simultaneously, reflecting the importance of intrinsic disorder in multifunctional proteins. Similarly, E3 ubiquitin-protein ligase TRIM36 (UniProt ID: Q9NQ86) comprised a serine protease domain and a C-type lectin domain within disordered regions, contributing to its function in the lectin pathway of the complement system. These multi-domain configurations suggest that proteins with multiple SCOP domains may exhibit complex functional roles, potentially influenced by the presence of intrinsic disorder within these domains. Such findings reinforce the idea that intrinsic disorder enhances versatility and adaptability in proteins that interact with diverse molecular partners.

Several superfamilies showed substantial disorder within individual proteins, highlighting the occurrence of intrinsic disorder within specific functional domains. For instance, the P-domain of calnexin/calreticulin superfamily had a high magnitude of disorder, with 70 disordered residues identified in the protein Calreticulin (UniProt ID: P27797). This domain’s disorder is likely crucial for its role in calcium binding and molecular chaperoning. Additionally, the Fibronectin type III domain, commonly involved in cell adhesion and interaction, exhibited 23 disordered residues and was present in protein Fibronectin type-III domain-containing protein 3A (UniProt ID: Q9Y2H6). This finding suggests that disorder in this domain may enhance flexibility and promote interactions with a range of cellular partners, further emphasizing the link between intrinsic disorder and functional plasticity.

The Ankyrin repeat superfamily also showed disorder, with 23 disordered residues found in the protein Neurogenic locus notch homolog protein 1 (UniProt ID: P46531), known for its role in protein–protein interactions. Other frequently disordered superfamilies included the ARM repeat (17 residues in Maestro heat-like repeat-containing protein family member 2B (UniProt ID: Q7Z745)) and the Sec1/munc18-like (SM) proteins (17 residues in Syntaxin-binding protein 1 (UniProt ID P61764)), both of which contribute to functional versatility in protein complexes. Collectively, these findings highlight how intrinsic disorder in SCOP domains supports functional diversity, particularly in proteins central to acrosomal processes.

**Figure 9:**
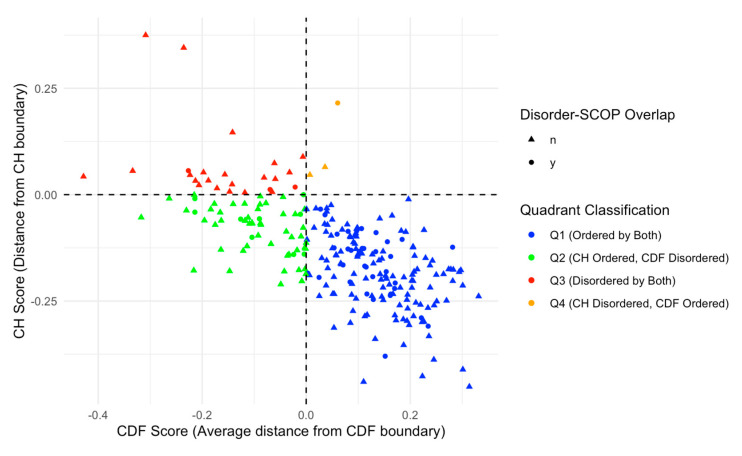
CH-CDF Plot with Disorder-SCOP Overlap. This plot illustrates the relationship between CH scores (distance from CH boundary) and CDF scores (average distance from CDF boundary) for proteins with and without disorder-SCOP overlaps. Data points are classified into four quadrants: Q1 (Ordered by both CH and CDF boundaries, blue), Q2 (CH Ordered, CDF Disordered, green), Q3 (Disordered by both CH and CDF boundaries, red), and Q4 (CH Disordered, CDF Ordered, orange). Circles (●) indicate proteins with disordered regions (classified by 75% agreement in disorder) that overlap with SCOP domains, while triangles (▲) indicate no overlap. This visualization highlights the distribution of structural classification across the disorder-CDF boundary space.

We further analyzed this subset of 48 proteins with SCOP domains within disordered regions by overlaying the data on the CH/CDF plot, as shown in Figure 9. This overlay provides a visual representation of the relationship between disorder predictions by Charge-Hydropathy and Cumulative Distribution Function metrics and the presence of disordered residues within SCOP domains. Of the 48 proteins with SCOP domains within the disordered region, 35 were in Q1 (ordered by both), 9 were in Q2 (CH ordered but CDF disordered), 3 were in Q3 (disordered by both), and 1 was in Q4 (CH disordered but CDF ordered).

### 3.4. Gene Ontology Enrichment Analysis of Acrosomal Proteins

**Figure 10:**
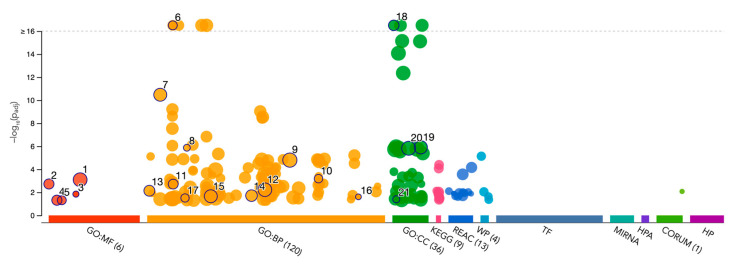
Gene Ontology Analysis of Disorder-Affected Proteins. This bubble plot displays enriched Gene Ontology (GO) terms for disorder-affected proteins across three GO categories: Molecular Function (GO:MF), Biological Process (GO:BP), and Cellular Component (GO:CC). The size of each bubble corresponds to the number of proteins associated with the term, while the y-axis represents the statistical significance (−log10 *p*-value). KEGG, Reactome (REAC), and other pathways are also included, illustrating the functional and structural roles of intrinsically disordered proteins within biological systems. Values above the dotted line threshold were capped.

**Figure 11:**
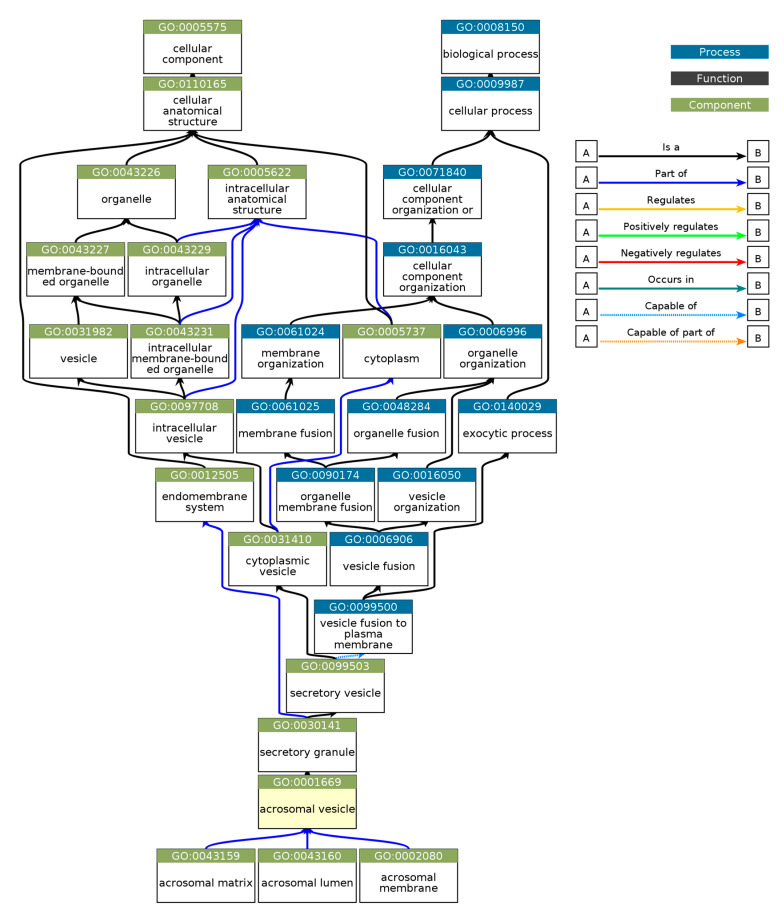
Gene Ontology (GO) Hierarchical Annotation for ‘Acrosomal Vesicle’ (GO:0001669). This hierarchical chart depicts the Gene Ontology relationships for GO:0001669 (acrosomal vesicle), a cellular component critical for acrosome structure and function during fertilization. This chart traces the acrosomal vesicle as part of higher-level biological processes, such as vesicle fusion (GO:0099500), organelle membrane fusion (GO:0090174), and membrane fusion (GO:0061025). These components are further nested under broad categories like organelle organization (GO:0006996) and cellular anatomical structure (GO:0110165). The acrosomal vesicle is functionally linked to structures like the acrosomal matrix (GO:0043159), acrosomal lumen (GO:0043160), and acrosomal membrane (GO:0002080), highlighting its role in vesicle organization and exocytosis. This chart was created using EMBL’s European Bioinformatics Institute’s QuickGO tool.

Gene Ontology (GO) enrichment analysis was performed using g:GOSt to understand the functional roles of disordered regions overlapping SCOP domains in acrosomal proteins (Table 3). The analysis provided insights into molecular functions, biological processes, and cellular components enriched among these proteins (Figure 10). Notably, the term ‘acrosomal vesicle’ (GO:0001669, p_adj = 5.395 × 10⁻³⁸) was the most significant, highlighting the functional relevance of intrinsic disorder in reproductive and cellular processes. The ancestor chart for ‘acrosomal vesicle’ (GO:0001669) is shown in Figure 11, as obtained from the European Bioinformatics Institute (https://www.ebi.ac.uk/QuickGO/ (accessed 27 October 2024)).

For molecular function, significant enrichment was observed for enzyme binding (GO:0019899), indicating that intrinsic disorder in these proteins may facilitate dynamic interactions with enzymatic partners, potentially critical for acrosomal activity. SNARE binding (GO:0000149) was also highly enriched, suggesting that these proteins play an integral role in membrane fusion events essential for the acrosomal reaction during fertilization. Additional enriched functions included cation binding and phospholipase activity, reflecting the importance of these proteins in calcium-mediated signaling and membrane remodeling within the acrosome.

Within the domain of biological processes, the most significant enrichment was observed for developmental processes involved in reproduction (GO:0003006), consistent with the essential role of these proteins in fertilization. Proteins with disordered regions were also enriched for acrosomal vesicle (GO:0098589), further emphasizing their association with the acrosome. Terms such as regulation of synaptic vesicle priming and cell projection organization were also enriched, highlighting their involvement in vesicle trafficking and cytoskeletal dynamics, both of which are processes critical for sperm functionality.

For cellular components, proteins were primarily localized to the acrosomal vesicle and the plasma membrane region, consistent with their roles in acrosomal membrane dynamics. Enrichment for the zonula adherens component suggested potential roles in adhesion, showing the interaction between the acrosome and the oocyte during fertilization.

These findings underscore the importance of intrinsic disorder in mediating the functional versatility of acrosomal proteins. The enrichment of terms associated with dynamic molecular interactions, membrane remodeling, and vesicular trafficking aligns with the known physiological roles of the acrosome in fertilization. The association of disordered regions with these processes suggests that intrinsic disorder contributes to the structural flexibility required for the complex interactions and functions of acrosomal proteins.

### 3.5. Interactivity Analysis of Human Acrosomal Proteins

**Figure 12:**
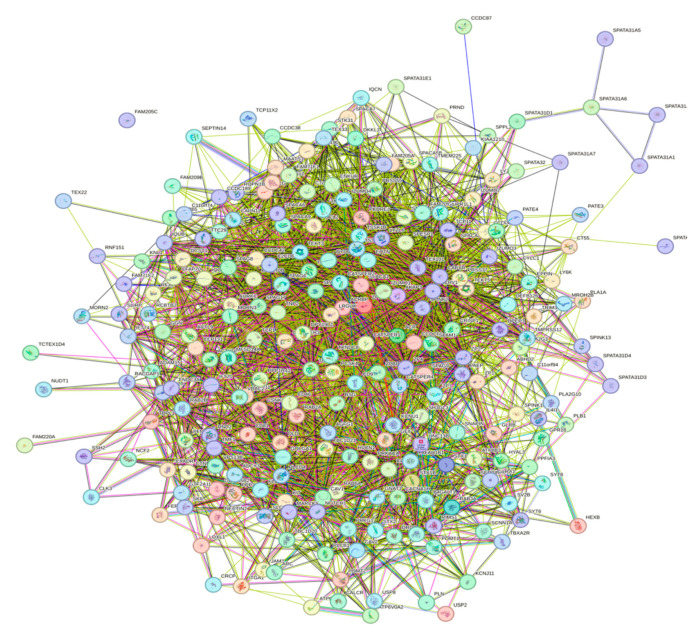
Inter-PPI Network of Human Acrosomal Proteins with 0.15 Confidence. STRING-based analysis of the inter-set interactivity of 245 human acrosomal proteins at the low confidence interval (0.15) to ensure maximum inclusion. Interactions are based on experimental and predicted information. All proteins except for one (UniProt ID: A6NFA0) have determined interactions.

A key feature of intrinsically disordered proteins is their high level of binding promiscuity and ability to interact with many partners through different binding modes [11]. The intractability of the human acrosomal proteins was investigated using Search Tool for the Retrieval of Interacting Genes (STRING). Figure 12 shows the PPI network for 245 of the human acrosomal proteins that were in the STRING database. No data were available in STRING for five of the proteins in our dataset: putative spermatogenesis-associated protein 31C1 (UniProt ID: P0DKV0), putative spermatogenesis-associated protein 31C2 (UniProt ID: B4DYI2), IQ domain-containing protein N (UniProt ID: Q9H0B3), spermatogenesis-associated protein 31D4 (UniProt ID: Q6ZUB0), and putative protein SPATA31F2P (UniProt ID: Q63HN1).

To capture the most interactions, a low confidence interval (0.15) was first utilized. The resulting PPI network showed that all 245 acrosomal proteins were interacting with each other, with the exception of one protein: protein SPATA31F3 (UniProt ID: A6NFA0). This PPI network comprised 245 nodes (human acrosomal proteins) connected by 2821 edges (PPIs). The average node degree (number of edges per node) was 23 and its average local clustering coefficient (which is a number between 0 and 1 that represents how complete the neighborhoods of interactions are, 0 meaning proteins in the neighborhood only connect to the same protein, and 1 meaning every protein in the neighborhood is interacting with each other) of 0.356. For a random group of proteins of the same size, the expected number of edges would be 942 at this confidence interval, which is significantly lower than our PPI network’s 2821, indicating at the low confidence level, this PPI network has roughly 3 times as many interactions as expected (PPI enrichment *p*-value of <10^−16^). This shows that most of the human acrosomal proteins are interacting with each other.

**Figure 13:**
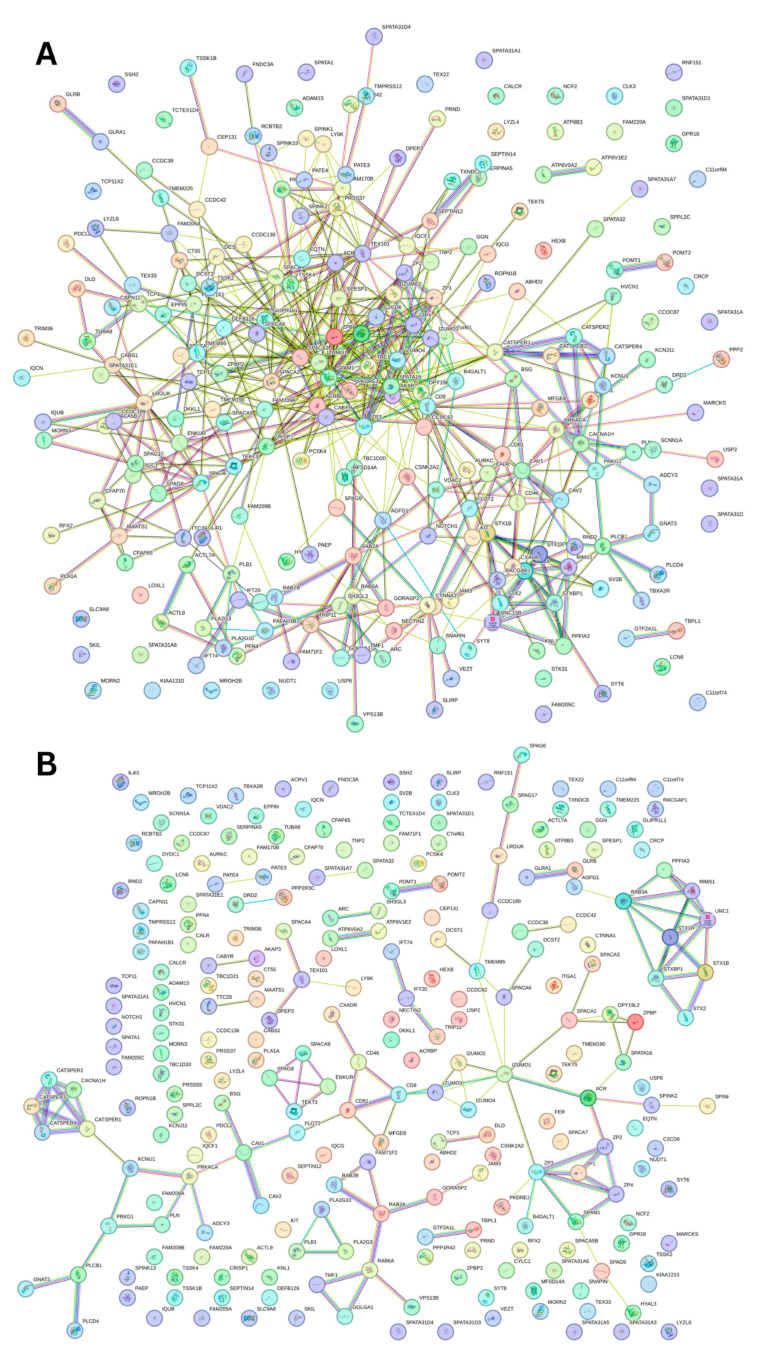
Inter-PPI Networks of Human Acrosomal Proteins with 0.4 and 0.7 Confidence. STRING-based analysis of the inter-set interactivity of 245 human acrosomal proteins at the (**A**) medium confidence interval (0.4) and (**B**) high confidence interval (0.7).

When the STRING confidence score was increased to a moderate threshold (0.4), the number of interacting nodes decreased from 244 to 209 (Figure 13A). The 209 nodes are connected by 581 edges, with an average node degree of 4.74, an average local clustering coefficient 0.423, an expected number of edges of 113, and a PPI enrichment *p*-value of <10^−16^. Increasing the confidence interval to high level (0.7) caused the PPI network to break down into small clusters of interacting proteins and many non-interacting proteins (Figure 13B).

**Figure 14:**
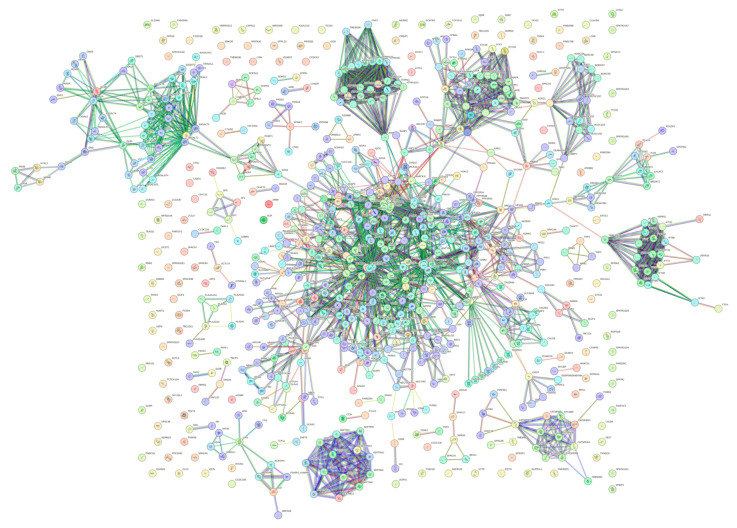
Global PPI Network at a 0.9 Confidence Interval. STRING-based analysis of 245 human acrosomal proteins with the 500 highest interacting proteins. The highest confidence was selected to see the most accurate interactions.

To explore the global interactivity of human acrosomal proteins, we incorporated their first shell of interactors—proteins that directly interact with our proteins of interest. These direct interactors are visualized in Figure 14. The PPI network was set to the highest confidence interval (0.9) and 500 interactants (the maximum value STRING allows) resulted in 745 nodes connected by 2523 edges, that is characterized by an average node degree of 6.77, and an average local clustering coefficient of 0.602. The expected number of edges for a PPI network of this size is 872, which indicates that the human acrosomal protein PPI network has significantly more interactions then expected with a PPI enrichment *p*-value of < 10^−16^.

### 3.6. Liquid–Liquid Phase Separation Propensity in Human Acrosomal Proteins

We used computational models to predict the liquid–liquid phase separation (LLPS) propensity for the 250 proteins in our acrosomal dataset. To quantify and understand LLPS trends, descriptive statistics for the LLPS propensity scores (p_LLPS_) are summarized in Table 4. The mean p_LLPS_ across all proteins was 0.4511, with a median of 0.2864 and a standard deviation of 0.3334. The scores ranged from 0.0922 to 1, with the lower quartile (Q1) at 0.167 and the upper quartile (Q3) at 0.7946. These findings indicate substantial variability in LLPS propensity among the acrosomal proteins.

To explore the relationship between intrinsic disorder and LLPS propensity, we conducted a regression analysis using the average PPIDR score from three disorder models (PONDR^®^ VXLT, PONDR^®^ VL3, and PONDR^®^ VSL2B) as the independent variable and p_LLPS_ as the dependent variable. A Box–Cox transformation of the p_LLPS_ scores (λ ≈ 0.0606) was applied to stabilize variance and improve linearity.

**Figure 15:**
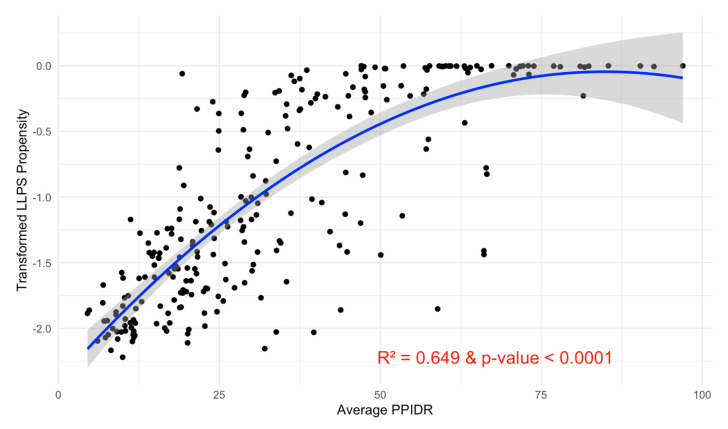
Relationship between Average PPIDR and Transformed p_LLPS_. This scatter plot illustrates the relationship between the average PPIDR of three models (x-axis) and transformed p_LLPS_ (y-axis). A fitted regression line with a 95% confidence interval (shaded region) shows a significant relationship. The model explains 64.9% of the variance (R^2^ = 0.649) and is highly significant (*p*-value < 0.0001).

The subsequent second-degree polynomial regression model revealed a significant positive relationship between intrinsic disorder and LLPS propensity, with a *p*-value < 0.0001 and an R-squared value of 0.649 (Figure 15). Diagnostic plots indicated that the model assumptions were satisfied, supporting the robustness of this relationship.

The observed link between intrinsic disorder and LLPS propensity aligns with the known role of intrinsically disordered regions (IDRs) in facilitating phase separation. IDRs, characterized by their lack of a fixed tertiary structure, enable dynamic interactions essential for condensate formation, a key feature of phase-separated systems. In the context of the acrosome, proteins with a high LLPS propensity likely contribute to the dynamic remodeling of the acrosomal vesicle and the assembly of molecular condensates critical for fertilization. For instance, proteins with high p_LLPS_ scores may facilitate rapid assembly and disassembly of protein complexes during the acrosome reaction, enabling precise spatiotemporal control over enzymatic and structural functions.

The wide range of p_LLPS_ scores suggests functional diversity within the acrosomal proteome. Proteins with low P_LLPS_ may perform structural roles, providing stability to the acrosomal matrix, while those with high p_LLPS_ likely play dynamic roles in processes such as vesicle fusion, membrane remodeling, and molecular recognition. This variability underscores the dual requirements for structural integrity and functional adaptability within the acrosome.

The polynomial nature of the relationship between PPIDR and p_LLPS_ highlights a nuanced interplay between intrinsic disorder and LLPS propensity. While higher levels of disorder generally enhance phase separation propensity, the saturation observed in the quadratic relationship suggests a threshold effect, where excessively disordered proteins may lack sufficient interaction interfaces for effective condensate formation. This balance may reflect evolutionary pressures to optimize disorder for LLPS functionality while maintaining the ability to form specific, transient interactions.

These findings provide a foundation for further investigation into the role of LLPS in acrosomal biology. Future studies could focus on experimentally validating the LLPS behavior of high-p_LLPS_ proteins using in vitro or in vivo approaches, such as fluorescence recovery after photobleaching (FRAP) or phase separation assays. Additionally, exploring the involvement of post-translational modifications (PTMs) in modulating LLPS propensity could provide insights into the regulation of acrosomal protein dynamics during fertilization.

### 3.7. Impact of Disorder and Liquid–Liquid Phase Separation in the Five Most Disordered Acrosomal Proteins

#### 3.7.1. Myristoylated Alanine-Rich C-Kinase Substrate Protein (UniProt ID: P29966)

**Figure 16:**
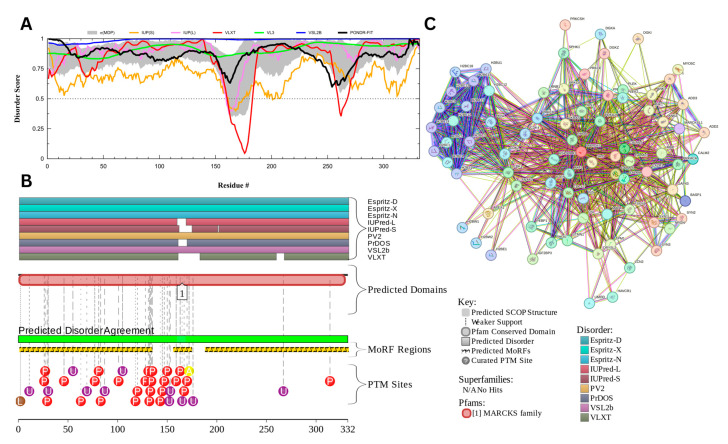
Functional Disorder Analysis of Myristoylated Alanine-Rich C-kinase Substrate (MARCKS) UniProt ID: P29966. (**A**) Intrinsic disorder profile generated by RIDAO. (**B**) Functional disorder profile generated by D^2^P^2^. (**C**) PPI network of MARCKS at 0.4 confidence interval.

The myristoylated alanine-rich C-kinase substrate (MARCKS) protein was identified as the most disordered among acrosomal proteins, with a percentage of predicted disordered residues of 97.0% ± 1.30% and an average disorder score of 0.890 ± 0.035. Figure 16 presents the complete computational analysis of the MARCKS protein. This protein is 332 amino acids long, and most of the residues show signs of disorder (Figure 16A). MARCKS has 1 Pfam conserved domain spanning from residue 2 to 329 corresponding to the MARCKS family. This protein also has multiple posttranslational modification sites spanning across the protein with most of them being in the first 176 residues and as well as three molecular recognition features associated with ANCHOR binding regions (Figure 16B). The PPI network (Figure 16C) generated by STRING for MARCKS at the medium confidence interval of 0.4 shows that the network comprises 81 nodes and 859 edges. The network has an average node degree of 21.2 interactions per node and the local clustering coefficient of 0.703. The expected number of edges is 393 and has a PPI enrichment *p*-value was highly significant (*p* < 10^−16^).

MARCKS has an important regulatory role in acrosomal exocytosis, mainly by modulating calcium mobilization and interacting with phosphatidylinositol 4,5-bisphosphate (PIP2) [155]. With a liquid–liquid phase separation (LLPS) propensity of 0.9995 (as per the FuzDrop analysis results), MARCKS likely contributes to the formation of dynamic, membrane-less compartments, which allow for spatial and temporal coordination of signaling molecules, which are essential for exocytosis. This high LLPS propensity and intrinsic disorder likelihood enables it to act as a molecular scaffold, holding PIP2 in concentrated, reversible phases that can be quickly disbanded upon phosphorylation by protein kinase C (PKC). The flexibility that the intrinsic disorder provides the MARCKS’ protein likely enhances its ability to form and dissolve these LLPS-driven condensates as needed, enabling the fast release of PIP2 for downstream signaling during acrosomal exocytosis [155,156]. This idea is in line with the observations that LLPS can be related to the orchestration of the kinase signaling representing a controllable mechanism of dynamic compartmentalization of kinases and their substrates and co-factors, as well as a potential means of the isolation of kinases from their inhibitors [157].

Multifunctionality and binding promiscuity of MARCKS show that this protein serves as an illustration of the aforementioned structure–function continuum model, where a protein exists as a dynamic conformational ensemble containing multiple proteoforms characterized by a broad spectrum of structural features and possessing various functional potentials. These proteoforms originate due to the presence of a large number of different PTMs, as well as because of the highly disordered status of this protein and structural changes induced by its functionality. Looking at the MARCKS from the perspective of a proteoform-based structure–function continuum helps better understanding of structure, regulation, and functionality of this important protein.

MARCKS’ disorder can be particularly important in its effector domain (ED), which interacts with PKC and calmodulin. When phosphorylated by PKC, MARCKS dissociates from the membrane, releasing PIP2 for phospholipase C (PLC) activity, which converts it to inositol trisphosphate (IP3) and diacylglycerol (DAG). This reaction is needed for initiating the release of calcium, a primary driver of membrane fusion in the acrosome reaction [158]. The high LLPS propensity of MARCKS supports a transient, phase-separated environment in which calcium-sensitive signaling molecules can be temporarily contained and released in a coordinated fashion. This shows its role as a highly dynamic regulatory scaffold within the sperm head. The ability of MARCKS to undergo phase separation could potentially streamline the availability of PIP2 and calcium within these condensates, which would emphasize the importance of structural reorganization to the functional requirements of the acrosomal exocytosis process [155].

Moreover, MARCKS’ ability to form LLPS-driven condensates can potentially be important for its interaction with actin, by cross-linking and stabilizing the cytoskeleton to prevent premature exocytosis under resting conditions. Upon PKC activation, MARCKS can change from a stabilizing role to a facilitative one, freeing calcium and reorganizing the actin filaments for exocytotic membrane fusion. This interaction between intrinsic disorder and LLPS likely enhances MARCKS’ function as a reversible regulator, with phosphorylation dictating its role and allowing specific control over acrosomal readiness and exocytotic response in fertilization [155].

#### 3.7.2. Nuclear Transition Protein 2 (UniProt ID: Q05952)

**Figure 17:**
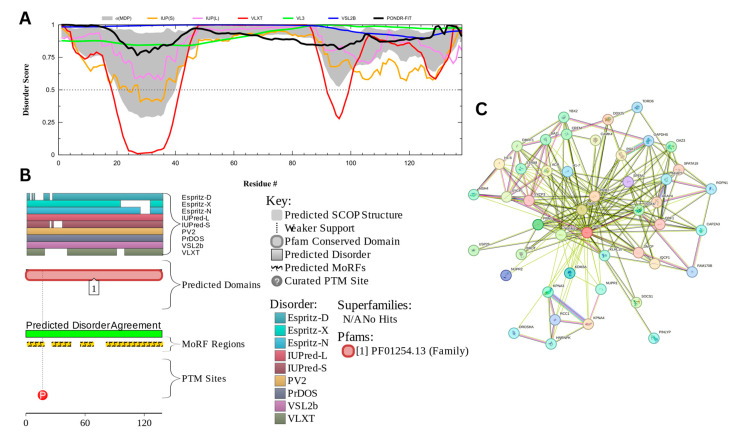
Functional Disorder Analysis of Nuclear Transition Protein 2 (TNP2) UniProt ID: Q05952. (**A**) Intrinsic disorder profile generated by RIDAO. (**B**) Functional disorder profile generated by D^2^P^2^. (**C**) PPI network of TNP2 at a 0.4 confidence interval.

Figure 17 presents the computational results of nuclear transition protein 2 or TNP2, which has a percentage of predicted disorder residues of 92.5% ± 3.22% and an average disorder score of 0.864 ± 0.036. This protein is 138 amino acids long all of which show signs of disorder (Figure 17A). TNP2 has 1 Pfam conserved domain spanning all 138 amino acids corresponding to the PF01254.13 family. This protein also has 1 posttranslational modification site at locus 17 and has 4 molecular recognition features associated with ANCHOR binding regions (Figure 17B). The PPI network (Figure 17C) generated by STRING for CEP131 at the medium confidence interval of 0.4 shows that the network comprises 46 nodes and 211 edges. The network has an average node degree of 9.17 interactions per node and the local clustering coefficient of 0.753. The expected number of edges is 51 and has a PPI enrichment *p*-value of less than 1.0 × 10^−16^.

TNP2 is an important protein involved in spermiogenesis. Its key function is facilitating chromatin remodeling through aiding in the removal of histones and taking their place within the chromatin before being replaced by protamines [159,160]. Studies have also shown that they are involved with DNA repair by preventing DNA breaks in late spermiogenesis [160]. Its ability to interact with diverse types of biologic molecules is likely facilitated by its high intrinsic disorder. The high probability of spontaneous liquid–liquid phase separation of 0.994 and the presence of two droplet-promoting regions (DPRs, residues 1–98 and 128–138) predicted by FuzDrop may be related to TNP2’s role of replacing histones, as histones have been found to have parts of their formation process linked to their LLPS [161]. Analysis of TNP2 localization patterns within the rat spermatid nucleus revealed that TNP2 is preferentially localized to the GC-rich DNA sequences, forming specific foci, some of which also contain TNP1 [162].

Clearly, this protein exists as a complex and highly dynamic conformational ensemble, in which one can find basic (or intrinsic, or conformational) proteoforms generated by the presence of intrinsic disorder, inducible (or modified) proteoforms generated by PTMs, and functioning proteoforms originating due to the structural alterations induced in a protein by interaction with its partners.

#### 3.7.3. Centrosomal Protein of 131 kDa (UniProt ID: Q9UPN4)

**Figure 18:**
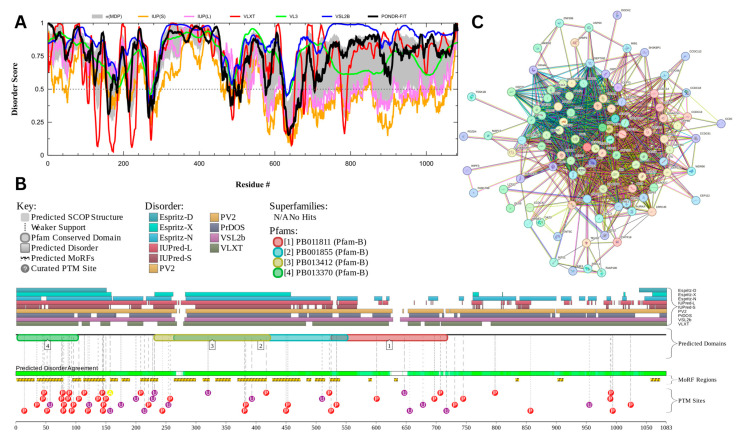
Functional Disorder Analysis of Centrosomal Protein of 131 kDA (CEP131). UniProt ID: (Q9UPN4) (**A**) Intrinsic disorder profile generated by RIDAO. (**B**) Functional disorder profile generated by D^2^P^2^. (**C**) PPI network of CEP 131 at a 0.4 confidence interval.

Figure 18 presents the results of centrosomal protein of 131 kDA (CEP131) which has a percentage of predicted disorder residues of 82.0% ± 7.35% and an average disorder score of 0.702 ± 0.043. This protein is 1083 amino acids long and has large areas of disorder spanning from N terminus to the C terminus (Figure 18A). CEP131 has 4 Pfam conserved domains spanning from Amino 3 to 104, 231 to 424, 264 to 553, and 526 to 719, respectively. This protein also has multiple posttranslational modification sites spanning across the protein with most of them being in the first 250 residues and has 20 molecular recognition features associated with ANCHOR binding regions (Figure 18B). It was pointed out that this protein has 3 isoforms produced by alternative splicing [163]. Therefore, in addition to the conformational and PTM-induced proteoforms, CEP131 has another type of inducible (or modified) proteoform, which is generated by alternative splicing. The PPI network (Figure 18C) generated by STRING for CEP131 at the medium confidence interval of 0.4 shows that the network comprises 101 nodes and 1392 edges. The network has an average node degree of 27.6 interactions per node and the local clustering coefficient of 0.735. The expected number of edges is 144 and has a PPI enrichment *p*-value of less than 10^−16^.

Centrosomal protein of 131 kDA (CEP131) is a protein that has a wide range of functions which may be linked to its high intrinsic disorder. CEP131 plays a key role in flagella and cilia formation as sperm cells lacking the proteins have malformed flagella [164,165]. Its ability to form flagella may be linked to interactions or a shared pathway CEP70 [165]. It has also been shown to interact with microtubule-based transport proteins such as dynein and could play a role in transport of material transport [166]. Its interactions with these microtubule proteins may also play a role in the formation of the acrosome as studies have shown mice lacking CEP131 have malformed sperm heads and fragmented acrosomes [164,167].

CEP131 has a high propensity for liquid–liquid phase separation (LLPS) with a probability of spontaneous liquid–liquid phase separation of 0.9989, suggesting that its significant intrinsic disorder and ability to form dynamic biomolecular condensates may facilitate its roles in flagella and acrosome formation, microtubule-based transport, and centrosome duplication. It was pointed out that in mice, male infertility is linked to the CEP131 loss that triggers defects in the manchette and flagella, indicating a possible link between CEP131 and the organization of cellular structures relying on LLPS [164]. Furthermore, another centrosomal protein, CEP112, was shown to undergo LLPS and form biomolecular condensates that recruit essential proteins and mRNAs [168].

Outside of its structural role in sperm, CEP131 plays a role in centrosome duplication and is up regulated in many cancers [169,170]. CEP131 is phosphorylated PIK4 with many of its phosphorylation sites located in its disordered regions. Once phosphorylated CEP 131 can recruit STIL which is a key protein for centrosome duplication [169]. CEP131 has also been found to interact with other regulatory genes such as ARID3A and NPM [170,171].

#### 3.7.4. Cylicin-1 (UniProt ID: P35663)

**Figure 19:**
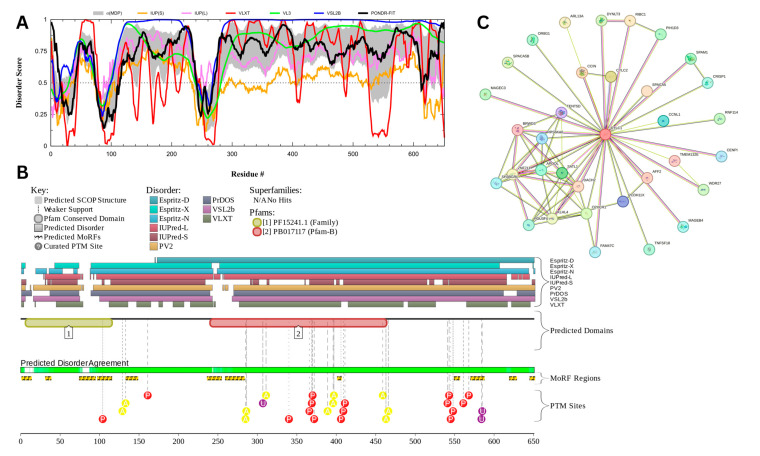
Functional Disorder Analysis of Cylicin-1 (CYLC1); UniProt ID: P35663. (**A**) Intrinsic disorder profile generated by RIDAO. (**B**) Functional disorder profile generated by D2P2. (**C**) PPI network of CYLC1 at a 0.4 confidence interval.

Figure 19 presents the computational analysis of Cylicin-1, or CYLC1, showing a predicted percentage of disordered residues at 81.1% ± 4.14% and an average disorder score of 0.745 ± 0.042. CYLC1 is a 651-amino-acid-long protein with most disorder localized toward the C-terminus starting at residue 268, while the N-terminal region shows smaller disordered segments (Figure 19A). This protein contains two Pfam conserved domains spanning amino acids 7–116 and 241–464, respectively. Multiple post-translational modification sites are clustered between residues 250 and 600, alongside 12 molecular recognition features (MoRFs) associated with ANCHOR-predicted binding regions (Figure 19B). The protein–protein interaction (PPI) network generated by STRING at a medium confidence interval (0.4) reveals 34 nodes and 75 edges, with an average node degree of 4.41 interactions per node, a local clustering coefficient of 0.877, and a statistically significant PPI enrichment (*p*-value = 4.84 × 10⁻^10^) (Figure 19C).

The high intrinsic disorder observed in CYLC1 likely facilitates its functional versatility, particularly in its role within the perinuclear theca (PT), a cytoskeletal element important for maintaining the acrosome’s attachment to the nucleus during spermiogenesis. CYLC1 has been shown to interact with itself and other PT proteins, such as ACTRT1, ACTL7A, and SPACA1, forming a structural ‘sandwich’ that anchors the acrosome to the nuclear envelope [172]. The disordered regions in CYLC1, particularly in the C-terminal domain, may enhance its capacity for multivalent interactions, allowing dynamic assembly and disassembly of protein complexes. This flexibility likely contributes to the stability of the acrosome–nucleus connection, as evidenced by the severe acrosome detachment and male subfertility observed in CYLC1 knockout mice [172].

Moreover, the clustering of MoRFs and post-translational modification (PTM) sites within the disordered regions of CYLC1 suggests a regulatory role in binding and interaction dynamics. These features may allow CYLC1 to act as a molecular scaffold, mediating the assembly of protein complexes that stabilize the acrosome’s structural integrity. PTMs such as phosphorylation could modulate the disorder-to-order transitions in these regions, dynamically regulating the protein’s interaction with binding partners during acrosomal maturation and exocytosis [117,172].

CYLC1’s intrinsic disorder might also influence its role in phase separation. The probability of spontaneous liquid–liquid phase separation of CYLC1 was 0.9965, which is incredibly high. The protein is also predicted by FuzDrop to contain 4 DPRs, residues 39–53, 101–245, 267–621, and 629–643. The enrichment of disordered and low-complexity regions makes CYLC1 a candidate for contributing to membraneless compartmentalization within the PT. Such phase-separated domains could act as reservoirs for critical proteins and molecular factors required for acrosomal function and fertilization, facilitating spatial and temporal coordination of these processes [118,172].

The STRING analysis further supports CYLC1’s role as a hub in the acrosomal interactome, with significant PPI enrichment and a high clustering coefficient indicating a tightly interconnected network of interactions. The observed disorder and network centrality together show CYLC1’s importance in ensuring the structural and functional integrity of the acrosome, with potential implications for male fertility in humans and animal models [172].

Similar to MARCKS and TNP2, multifunctionality and binding promiscuity of CYLC1 are determined by its highly disordered nature and the presence of numerous PTMs, generating basic and inducible proteoforms, respectively.

#### 3.7.5. Coiled-Coil Domain-Containing Protein 136 (CCDC136) (UniProt ID: Q96JN2)

**Figure 20:**
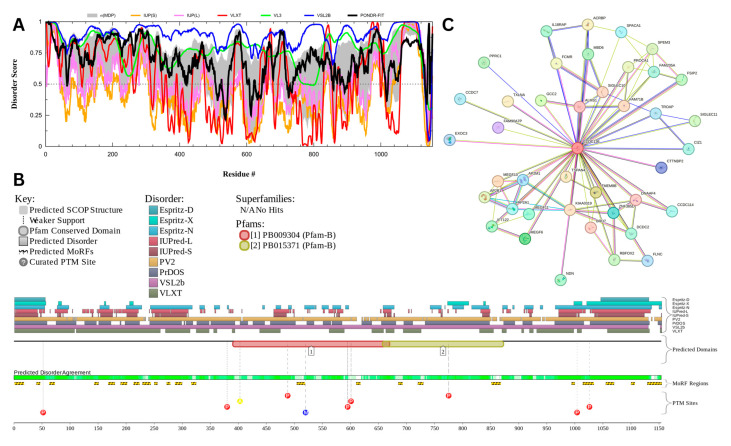
Functional Disorder Analysis of Coiled-Coil Domain-Containing Protein 136 (CCDC136); UniProt ID: Q96JN2. (**A**) Intrinsic disorder profile generated by RIDAO. (**B**) Functional disorder profile generated by D2P2. (**C**) PPI network of CCDC136 at a 0.4 confidence interval.

Figure 20 presents the computational analysis of CCDC136, showing a predicted percentage of disordered residues at 72.9% ± 10.02% and an average disorder score of 0.639 ± 0.062. CCDC136 is a 1154-amino-acid-long protein with disordered regions distributed throughout its sequence (Figure 20A). The protein contains two Pfam conserved domains spanning residues 391–670 and 658–873, respectively, and features 22 molecular recognition features (MoRFs) predicted by ANCHOR. In addition, CCDC136 has multiple post-translational modification (PTM) sites scattered across its sequence, further highlighting its potential regulatory complexity (Figure 20B). Furthermore, complexity of this protein is further increased by the presence of four isoforms generated by alternative splicing, where the isoform #2 is different from the canonical form by changing the 922–957 region from IKELQTKLRELQLQYQASMDEQGRLLVVQEQLEGQL to NMFGLWKPMVFLAIAAVA LYVLPNMRQQESEFCLME and missing the 958–1154 region [163], the isoform #3 has an MEAGAGAGAGAAGWSCPGPGPTVTTLGSYEASEGCERKKGQRWGSLERRGM extension at N-terminus and is missing regions 212–223 and 364–1121 [163], and the isoform #4, being associated with gastric cancer, is characterized by changes in the 1122–1154 region from SSPTPNPPIFSLPLVGLVVISALLWCWWAETSS to NMFGLWKPMVFLAIAAVALYVLPNMRQQESEFCLME [173]. The protein–protein interaction (PPI) network generated by STRING at a medium confidence interval of 0.4 (Figure 20C) shows that CCDC136 interacts with 41 nodes via 86 edges, with an average node degree of 4.2 interactions per node, a local clustering coefficient of 0.735, and a statistically significant PPI enrichment (*p*-value < 1.0 × 10⁻¹⁶). Its predicted liquid–liquid phase separation propensity (p_LLPS_) is high, at 0.9894, suggesting a strong potential for forming biomolecular condensates.

The intrinsic disorder of CCDC136 likely plays a key role in mediating its function during acrosome biogenesis and fertilization. Studies indicate that CCDC136 localizes to the acrosome during spermatogenesis, where it is critical for the correct assembly of this specialized organelle [174]. The protein’s disordered regions may provide the flexibility required for dynamic interactions with other acrosome-related proteins, such as SPACA1 and PICK1, which are involved in the early stages of acrosome formation and are significantly downregulated in CCDC136 knockout mice [174]. The distribution of MoRFs across CCDC136 supports its role as a scaffold that mediates transient, multivalent interactions essential for acrosomal maturation.

CCDC136’s high p_LLPS_ score (0.9894) and the presence of multiple DPRs (residues 1–55, 70–99, 149–170, 195–227, 240–288, 303–320, 518–531, 789–800, 832–845, 908–928, and 967–1143) further suggests its involvement in forming liquid-like condensates within the acrosome. These condensates may act as compartments for concentrating acrosome-specific proteins, ensuring their correct localization and function during spermiogenesis. The ability to undergo phase separation could also help stabilize the structural integrity of the acrosome and facilitate its rapid reorganization during the acrosome reaction, a process critical for successful fertilization [118]. In CCDC136 knockout mice, the disruption of acrosome morphology and the associated male infertility underscore the importance of this protein’s role in both structural and functional aspects of spermatogenesis [174].

Moreover, the presence of PTMs within disordered regions of CCDC136 provides an additional regulatory layer, potentially controlling the protein’s interactions and phase-separation behavior. PTMs such as phosphorylation may modulate CCDC136’s role in acrosome formation by dynamically altering its binding affinity to partner proteins or its ability to assemble into condensates. This aligns with the broader understanding of disordered proteins as dynamic regulators in cellular processes, where disorder and phase separation allow for reversible, context-dependent assembly of protein complexes [117].

The STRING network further illustrates CCDC136’s centrality in the acrosomal protein interaction network, with its significant enrichment of interactions indicating a pivotal role in coordinating processes necessary for spermatogenesis and fertilization. The interplay between CCDC136’s intrinsic disorder, conserved domains, and its ability to form condensates highlights its dual role as both a structural and regulatory component of the acrosome. Again, the proteoform-based structure–function continuum model provides mechanistic explanation of the multifunctionality of CCDC136 and its ability to interact with multiple partners and control their functionality.

#### 3.7.6. Coordinated Roles of Highly Disordered Acrosomal Proteins in Fertility

Summarizing, our analyses of the most disordered acrosomal proteins provide important clues on the links between their functionality, intrinsic disorder propensity, and LLPS potential.

MARCKS, with its important regulatory role in acrosomal exocytosis—the central event of the acrosome reaction—is a highly disordered protein (PPIDR = 97.0% ± 1.30%; ADS = 0.890 ± 0.035) and exhibits an exceptionally high LLPS propensity (p_LLPS_ = 0.9995). TNP2 (PPIDR = 92.5% ± 3.22%; ADS = 0.864 ± 0.036; p_LLPS_ = 0.9440) plays a key role in sperm chromatin remodeling by facilitating the removal of histones and their replacement within the chromatin, prior to being displaced by very alkaline protamines. This process, known as sperm chromatin protamination, is fundamental to sperm maturation and leads to the tightly compacted toroidal chromatin architecture of the sperm head, which is essential for fertilization. Centrosomal protein CEP131 (PPIDR = 82.0% ± 7.35%; ADS = 0.702 ± 0.053; p_LLPS_ = 0.9989) is critical for flagella formation and thus, sperm motility. Importantly, both acrosome reaction and motility activation are coordinated responses triggered during zona pellucida (ZP) penetration and are regulated by progesterone and FSH produced by the egg and surrounding cumulus cells. CYLC1 (PPIDR = 81.1% ± 4.14%; ADS = 0.745 ± 0.042; p_LLPS_ = 0.9965), which functions in anchoring the acrosome to the sperm nucleus, also serves as a highly connected hub within the acrosome interactome of intrinsically disordered proteins.

These observations highlight how highly disordered acrosomal proteins, beyond their direct roles in acrosomal exocytosis and LLPS, contribute to core processes essential to sperm fertility—including chromatin remodeling and flagella formation. Collectively, they underscore the multifunctionality conferred by intrinsic disorder, which enables these proteins to coordinate diverse molecular events that are fundamental to human fertility and spermiogenesis.

## 4. Conclusions

Our study investigated the role of intrinsic disorder and liquid–liquid phase separation (LLPS) in the human acrosomal proteome through bioinformatics and computational analysis. This analysis shows that approximately 81% of the 250 human acrosomal proteins in our study had signs of moderate to high levels of disorder, and the flexibility of these disordered regions may aid their roles in fertilization. These disordered and LLPS-prone regions likely enhance acrosomal protein function by enabling rapid, multivalent interactions and dynamic molecular assembly, which are critical for processes like vesicle fusion, enzymatic release, and sperm-egg membrane interactions during fertilization. This is further supported by the fact that the most common SCOP superfamilies found among the disordered regions were associated with structural flexibility and protein–protein interactions, which are hallmarks of IDPs and IDRs. The STRING analysis revealed a prominent level of interaction between the acrosomal proteins and other proteins showing their high levels of promiscuity. When looking into the LLPS propensity, 30% of the acrosomal proteins were high-probability LLPS drivers. In the context of the acrosome, the proteins with high LLPS propensity contribute to the acrosomal vesicle and the assembly of molecular condensates critical for fertilization.

While our findings highlight the significance of intrinsic disorder and LLPS in the acrosomal proteome, it is also important to recognize that ordered proteins play essential roles in fertilization as well, and a comprehensive understanding of acrosomal function should consider both structured and disordered components.

Results of our study add a new perspective to the functionality and formation of the human acrosome through the identification of IDPs and IDRs, and LLPS within its proteome. These proteins with high intrinsic disorder and LLPS propensity, such as myristoylated alanine rich C-kinase substrate (MARCKS; UniProt ID: P29966) and nuclear transition protein 2 (TNP2; UniProt ID: Q05952) could serve as potential targets for understanding acrosome deformities and infertility. Given growing global concerns about declining male fertility [4], understanding the roles of intrinsically disordered and LLPS-prone proteins in acrosomal structure and function may not only advance basic reproductive biology but also hold applied value for developing diagnostic or predictive tools in assisted reproduction.

## Figures and Tables

**Table 1:** Summary Statistics for ADS and PPIDR Across PONDR^®^ VXLT, PONDR^®^ VSL2B, and PONDR^®^ VL3 Models. This table presents the minimum, mean, median, and maximum values for ADS (average disorder score) and PPIDR (percent predicted intrinsically disordered residues) for three disorder prediction models: PONDR^®^ VXLT, PONDR^®^ VSL2B, and PONDR^®^ VL3.

	PONDR^®^ VXLT		PONDR^®^ VSL2B		PONDR^®^ VL3	
	ADS	PPIDR	ADS	PPIDR	ADS	PPIDR
Minimum	0.0602	0.79	0.1755	4.79	0.0995	0
Mean	0.3435	32.09	0.4582	40.83	0.3999	33.37
Median	0.3177	28.88	0.4141	34.04	0.3693	27.14
Maximum	0.8295	90.96	0.9907	100	0.9240	100

**Table 2:** SCOP Superfamilies Affected by Disorder and Associated Proteins. This table lists SCOP superfamilies impacted by intrinsic disorder, including the total number of residues affected and the corresponding associated proteins. This highlights the overlap between structural disorder and SCOP domain regions.

Superfamily	Disordered Residues (n)	Proteins Affected (UniProt IDs)
ARM repeat	17	Q7Z745
Actin-like ATPase domain	2	Q9Y615
Ankyrin repeat	23	P46531
C-terminal domain of PLC-beta	40	Q9NQ66
C2 domain (Calcium/lipid-binding domain, CaLB)	14	O14795
CAD and PB1 domains	15	P19878
CATH	1	P17612
Calpain large subunit, middle domain (domain III)	3	Q9UMQ6
Concanavalin A-like lectins/glucanases	40	P27797, Q9NQ86
Cysteine proteinases	13	O75604
Cysteine-rich domain	4	Q9H0H5
Dimerization-anchoring domain of cAMP-dependent PK regulatory subunit	4	Q9BZX4
EF-hand	1	Q9NQ66
FAD-linked reductases, C-terminal domain	1	Q96RQ9
FAD/NAD(P)-binding domain	3	Q96RQ9
FYVE/PHD zinc finger	36	Q86UR5
Family A G protein-coupled receptor-like	5	P14416, P21731
Fibronectin type III	23	Q9Y2H6
Growth factor receptor domain	6	Q6UW60
HRDC-like	17	O75575
Immunoglobulin	3	P35613, Q92692
Integrin domains	4	P56199
MIR domain	5	Q9UKY4, Q9Y6A1
Neurotransmitter-gated ion-channel transmembrane pore	40	P23415, P48167
Nucleotide cyclase	10	O60266
Nucleotide-diphospho-sugar transferases	12	P15291
P-domain of calnexin/calreticulin	70	P27797
P-loop containing nucleoside triphosphate hydrolases	9	P20340
PDZ domain-like	10	Q86UR5
PLC-like phosphodiesterases	111	Q9BRC7, Q9NQ66
Phospholipase A2, PLA2	15	Q9NZ20
Protein kinase-like (PK-like)	31	Q13976, Q9UQB9
Rhodanese/Cell cycle control phosphatase	7	P40818
SGNH hydrolase	6	Q6P1J6
SH3-domain	24	P19878
Sec1/munc18-like (SM) proteins	17	P61764
Serpins	5	P05154
Subtilisin-like	11	Q6UW60
Thioredoxin-like	4	Q8N4E4
Transcription factor IIA (TFIIA), β-barrel domain	6	Q9UNN4
Trypsin-like serine proteases	11	Q6UWB4
Tubulin C-terminal domain-like	5	Q9NY65
USP8 N-terminal domain-like	20	P40818
WD40 repeat-like	10	P43034
Ypt/Rab-GAP domain of gyp1p	8	Q96BZ9
alpha-catenin/vinculin-like	10	P35221
t-SNARE proteins	73	P32856, P61266, Q16623

**Table 3:** Enriched Gene Ontology (GO) Terms for Disorder-Affected Proteins. This table lists enriched GO terms across Molecular Function (GO:MF), Biological Process (GO:BP), and Cellular Component (GO:CC) categories. Each row includes the GO Term ID, name, and the adjusted *p*-value (p_adj), indicating the statistical significance of enrichment. Notably, terms such as ‘acrosomal vesicle’ (GO:0001669, p_adj = 5.395 × 10⁻^38^) highlight the functional relevance of intrinsic disorder in reproductive and cellular processes.

Source	Term ID	Term Name	p_adj (Query)
GO:MF	GO:0019899	enzyme binding	7.900 × 10^−4^
	GO:0000149	SNARE binding	1.876 × 10^−3^
	GO:0016934	extracellularly glycine−gated chloride channel …	1.430 × 10^−2^
	GO:0043169	cation binding	4.442 × 10^−2^
	GO:0005484	SNAP receptor activity	4.490 × 10^−2^
	GO:0004620	phospholipase activity	4.815 × 10^−2^
GO:BP	GO:0003006	developmental process involved in reproduction	3.127 × 10^−11^
	GO:0010807	regulation of synaptic vesicle priming	1.386 × 10^−6^
	GO:0065008	regulation of biological quality	9.343 × 10^−6^
	GO:0099170	postsynaptic modulation of chemical synaptic t …	6.720 × 10^−4^
	GO:0007405	neuroblast proliferation	1.993 × 10^−3^
	GO:0048870	cell motility	5.806 × 10^−3^
	GO:0000910	cytokinesis	7.271 × 10^−3^
	GO:0045787	positive regulation of cell cycle	2.231 × 10^−2^
	GO:0031175	neuron projection development	2.257 × 10^−2^
	GO:1904100	positive regulation of protein O−linked glycosyl …	2.547 × 10^−2^
	GO:0010560	positive regulation of glycoprotein biosynthet …	3.189 × 10^−2^
GO:CC	GO:0001669	acrosomal vesicle	5.395 × 10^−38^
	GO:0098590	plasma membrane region	1.175 × 10^−6^
	GO:0042995	cell projection	1.406 × 10^−6^
	GO:0005915	zonula adherens	3.998 × 10^−2^

**Table 4:** LLPS Propensity Summary. Summary of descriptive statistics for the LLPS propensity of proteins, including measures of central tendency (mean and median), variability (standard deviation), and distribution (minimum, maximum, and quartiles).

Statistic	LLPS Propensity Statistics
Count (N)	250
Mean (x¯)	0.451146
Std. Deviation (σ)	0.33337186
Minimum (x_min_)	0.0922
First Quartile (Q1))	0.166975
Median (Q2)	0.28645
Third Quartile (Q3)	0.7946
Maximum (x_max_)	1

## Data Availability

Data are contained within this article.

## Abbreviations

The following abbreviations are used in this manuscript:

ADS	Average Disorder Score
ANN	Artificial Neural Network
ATP	Adenosine Triphosphate
CDF	Cumulative Distribution Function
CEP	Centrosomal Protein
CH	Charge-Hydropathy
Cryo-EM	Cryo-Electron Microscopy
D2P2	Database of Disordered Protein Predictions
DAG	Diacylglycerol
DPR	Droplet Promoting Regions
FDR	False Discovery Rate
FRAP	Fluorescence Recovery After Photobleaching
GO	Gene Ontology
IAM	Inner Acrosomal Membrane
IDP	Intrinsically Disordered Protein
IDR	Intrinsically Disordered Region
IP3	Inositol Trisphosphate
KEGG	Kyoto Encyclopedia of Genes and Genomes
LLPS	Liquid–Liquid Phase Separation
MARCKS	Myristoylated Alanine-Rich C-Kinase Substrate
MoRF	Molecular Recognition Feature
NMR	Nuclear Magnetic Resonance
OAM	Outer Acrosomal Membrane
PIP2	Phosphatidylinositol 4,5-Bisphosphate
PKC	Protein Kinase C
PLC	Phospholipase C
pLDDT	Predicted Local Distance Difference Test
pLLPS	Probability of Liquid–Liquid Phase Separation
PONDR	Predictor of Natural Disordered Regions
PPI	Protein–Protein Interaction
PPIDR	Percentage of Predicted Intrinsically Disordered Residues
PTM	Post-Translational Modification
Q–Q	Quantile−Quantile
RIDAO	Rapid Intrinsic Disorder Analysis Online
SBIND	Sequence-Based Interaction Mode Divergence
SCOP	Structural Classification of Proteins
SNARE	Soluble N-ethylmaleimide-Sensitive Factor Attachment Protein Receptor
SVM	Support Vector Machine
TNP2	Nuclear Transition Protein 2
ZP	Zona Pellucida
